# GWAS identifies a molecular marker cluster associated with monoterpenoids in grapes

**DOI:** 10.1093/hr/uhaf144

**Published:** 2025-06-09

**Authors:** Hui-Min Zhang, Xin-Jie Lyu, Zheng-Yang Sun, Qi Sun, Ya-Chen Wang, Lei Sun, Hai-Ying Xu, Lei He, Chang-Qing Duan, Qiu-Hong Pan

**Affiliations:** Center for Viticulture and Enology, College of Food Science and Nutritional Engineering, China Agricultural University,17 Qinghuadonglu, HaiDian District, Beijing 100083, China; Key Laboratory of Viticulture and Enology, Ministry of Agriculture and Rural Affairs, 17 Qinghuadonglu, HaiDian District, Beijing 100083, China; Center for Viticulture and Enology, College of Food Science and Nutritional Engineering, China Agricultural University,17 Qinghuadonglu, HaiDian District, Beijing 100083, China; Key Laboratory of Viticulture and Enology, Ministry of Agriculture and Rural Affairs, 17 Qinghuadonglu, HaiDian District, Beijing 100083, China; Center for Viticulture and Enology, College of Food Science and Nutritional Engineering, China Agricultural University,17 Qinghuadonglu, HaiDian District, Beijing 100083, China; Key Laboratory of Viticulture and Enology, Ministry of Agriculture and Rural Affairs, 17 Qinghuadonglu, HaiDian District, Beijing 100083, China; Center for Viticulture and Enology, College of Food Science and Nutritional Engineering, China Agricultural University,17 Qinghuadonglu, HaiDian District, Beijing 100083, China; Key Laboratory of Viticulture and Enology, Ministry of Agriculture and Rural Affairs, 17 Qinghuadonglu, HaiDian District, Beijing 100083, China; Center for Viticulture and Enology, College of Food Science and Nutritional Engineering, China Agricultural University,17 Qinghuadonglu, HaiDian District, Beijing 100083, China; Key Laboratory of Viticulture and Enology, Ministry of Agriculture and Rural Affairs, 17 Qinghuadonglu, HaiDian District, Beijing 100083, China; Institute of Forestry and Pomology, Beijing Academy of Forestry and Pomology Sciences,12 Ruiwang Tomb, HaiDian District, Beijing 100093, China; Institute of Forestry and Pomology, Beijing Academy of Forestry and Pomology Sciences,12 Ruiwang Tomb, HaiDian District, Beijing 100093, China; Center for Viticulture and Enology, College of Food Science and Nutritional Engineering, China Agricultural University,17 Qinghuadonglu, HaiDian District, Beijing 100083, China; Key Laboratory of Viticulture and Enology, Ministry of Agriculture and Rural Affairs, 17 Qinghuadonglu, HaiDian District, Beijing 100083, China; Center for Viticulture and Enology, College of Food Science and Nutritional Engineering, China Agricultural University,17 Qinghuadonglu, HaiDian District, Beijing 100083, China; Key Laboratory of Viticulture and Enology, Ministry of Agriculture and Rural Affairs, 17 Qinghuadonglu, HaiDian District, Beijing 100083, China; Center for Viticulture and Enology, College of Food Science and Nutritional Engineering, China Agricultural University,17 Qinghuadonglu, HaiDian District, Beijing 100083, China; Key Laboratory of Viticulture and Enology, Ministry of Agriculture and Rural Affairs, 17 Qinghuadonglu, HaiDian District, Beijing 100083, China

## Abstract

Monoterpenoids are vital compounds that impart a distinctive floral flavor. They exist in both glycosidic and free forms in grapes. The breeding of improved monoterpenoid varieties has consistently been a topic of interest, yet only a limited number of molecular markers have been documented. This study employed a genome-wide association study (GWAS) on an F_1_ population crossed between a typical muscat variety (‘Muscat of Alexandria’) and a non-aromatic variety (‘Christmas Rose’), conducted over two consecutive years. A total of 4089 significant single nucleotide polymorphism sites (sigSNPs) and 892 candidate genes associated with monoterpenoids were identified. The sigSNPs corresponding to the glycosidic and total (glycosidic plus free) concentrations of various monoterpenoid compounds exhibited a high similarity. The majority of sigSNPs were located on chromosome 5, indicating the existence of a monoterpenoid-related marker cluster. Sixty-one lead SNPs located within the gene region and stably appearing in 2 years were selected and verified using a germplasm population. The alleles of the 25 lead SNPs were confirmed to be highly associated with monoterpenoid levels. The genes containing these lead SNPs were mainly glycoside hydrolase, ABC transporter, as well as the previously reported 1-deoxy-D-xylulose-5-phosphate synthase (*VvDXS1*) and geranylgeranyl pyrophosphate synthase large subunit (*VvGGPPS-LSU*). The function of VvGGPPS-LSU in regulating monoterpenoid levels was elucidated through *in vivo* overexpression, demonstrating the reliability of the marker cluster. The present study proposes a molecular marker set for the breeding with the objective of improving aroma, and a candidate gene network for the regulation of monoterpenoid synthesis in grapevine.

## Introduction

The grape (*Vitis vinifrea* L*.*) is a globally popular fruit, largely due to its unique flavor and perceived healthy benefits [1, 2]. Among the attributes that define grape quality, aroma is a critical quality trait influencing consumer preference and market value. The complex bouquet of grape aroma is composed of a diverse array of volatile compounds, including green leaf volatiles, benzenoids, esters, norisoprenoids, and terpenoids [3]. Among these, monoterpenoids stand out for their essential role in imparting floral, sweet, and citrus scents to aromatic grape varieties, such as ‘Muscat Ottonel’ and ‘Gewurztraminer’ [4–6]. These compounds are generally described as the key contributors to the characteristic ‘Muscat’ flavor, which is highly desirable in fresh grapes and grape-derived products like wine and juice.

Over 40 distinct monoterpenoid compounds have been identified in grapes [6], including alcohols (e.g. linalool, geraniol, nerol), hydrocarbons (e.g. *α*-terpinene, *β*-myrcene, ocimene), and derivatives (e.g. linalool oxide, rose oxide). In grape berries, monoterpenoids exist in both free and glycosidic forms, with the latter serving as important precursors for wine aroma [7]. Although glycosidic monoterpenoids are nonvolatile and do not directly contribute to the olfactory profile, they can be hydrolyzed during winemaking, releasing odorous aglycones that enhance the aromatic complexity of wine [8]. Typically, glycosidic monoterpenoids are present at higher concentrations than their free forms, making them a critical component in evaluating overall monoterpenoid synthesis in grapes [9]. Despite their significance, glycosidic monoterpenoids have historically received less attention in research compared to their free counterparts.

The genetic and transcriptional regulation of monoterpenoid metabolism has been the subject of numerous studies. Monoterpenoids are synthesized via the 2-C-methyl-D-erythritol-4-phosphate (MEP) pathway [10, 11], with 1-deoxy-d-xylulose 5-phosphate synthase (*DXS*) identified as a rate-limiting enzyme [12]. Terpene synthases (*TPS*s) play a direct role in the synthesis of terpenoid compounds, and at least 69 functional *TPS* genes have been predicted in the grapevine genome [13]. However, functional characterization of these genes remains limited [13–16]. The formation of monoterpenoid derivatives through oxidation, reduction, and glycosylation involves numerous enzymes, many of which are still poorly understood. For example, VvCYP76F14 is the only enzyme known to be involved in the transformation from linalool to wine lactone precursor [17], while only a few genes responsible for monoterpenol glycosylation have been identified, such as *VvGT7*, *VvGT14*, and *VvUGT85A* [18–20]. Recent studies have also identified transcription factors (e.g. *VvERF003* and *VvWRKY40* [21–23]) that regulate the expression of *VvGT14*. Despite these advances, the genetic regulation of monoterpenoid synthesis in grapes remains largely unexplored.

The content of monoterpenoids, as a quantitative trait determined by multiple minor genes and environmental factors, complicates efforts to develop genetic markers for its breeding. Previous studies have identified specific mutations K284N associated with increased monoterpenoid content in grapes through increasing the catalytic efficiency of VvDXS1 [24, 25]. This marker has been applied to grape aroma breeding [26]. Similar approaches have been successful in other plants, such as identifying alleles linked to geraniol content in roses [27]. In eucalyptus, GWAS identified an SNP locus (Chr01_25455245) highly associated with *β*-pinene levels [28]. These findings highlight the potential of quantitative trait locus (QTL) and GWAS in uncovering genetic markers for monoterpenoid traits. Nevertheless, most of these markers remain unverified, and there is a need for advanced techniques to identify additional genetic loci and candidate genes. GWAS provides a powerful tool for elucidating the molecular markers and causative genes of complex traits at the single-base level. This technique has been successfully applied in grapevine to identify genetic loci associated with traits such as seed size, skin color, berry weight, firmness, texture, and resistance to biotic or abiotic stresses [29–34]. Leveraging GWAS and molecular biology tools will be critical for unraveling the genetic architecture of monoterpenoid biosynthesis and advancing marker-assisted breeding strategies in grapevines.

In this study, we conducted a GWAS on a hybrid population to identify molecular markers and candidate genes associated with monoterpenoid levels. Unlike previous studies that focused primarily on free-form monoterpenoids, we investigated both the glycosidic-bound forms and the total concentrations (sum of glycosidic and free forms) of various monoterpenoid components. To ensure the reliability of the GWAS results, we validated the candidate SNP markers in a diverse germplasm population comprising 97 *Vitis* species. Based on these results, we constructed a network of candidate genes related to various monoterpenoid traits and further elucidated the function of a novel candidate gene, VvGGPPS-LSU. This study provides a validated marker cluster directly applicable for molecular breeding of aroma-optimized cultivars and enhances our understanding of the regulatory mechanisms underlying monoterpenoid biosynthesis in grapes, with particular emphasis on the previously overlooked glycosidic forms.

## Results

### Variation of monoterpenoid concentrations in F_1_ hybrid population

The monoterpenoids present in the two parents were characterized and quantified. The ‘Christmas Rose’ (CR) grapes were found to contain only linalool and geraniol, whereas the ‘Muscat of Alexandria’ (MA) grapes exhibited the presence of 15 distinct monoterpenoid compounds. These included five cyclic monoterpenoids, eight chain monoterpenoids, and two oxidized monoterpenoids (Table S1). Monoterpenoids were present in both their free and glycosidic forms. The concentration of monoterpenoids in the free form was lower than that of their glycosidic form. The most abundant compounds were *β*-myrcene and *β*-ocimene, followed by linalool and geraniol. The four compounds exhibited the average levels >10 μg/l, while the oxidized monoterpenoids were present at levels <1 μg/l.

A total of 126 and 116 individuals from the hybrid population were investigated in 2017 and 2018, respectively. Fifteen and 13 monoterpenoid compounds were identified in the 2 years, respectively. Notably, *γ*-terpinene and rose oxide were not detected in 2018 (Tables S1 and S2). The majority of compounds in the F_1_ population exhibited a greater concentration range in 2018 than in 2017, which may be attributed to the climatic differences between the 2 years. The compounds were present in both free and glycosidic forms, and the sum of them was designed as the total form. The compounds *γ*-terpinene and citronellol in 2017, along with hotrienol in both 2017 and 2018, were exceptions. They were exclusively detected in their free forms, likely because their glycosidic forms fell below the quantification limit. Specifically, *γ*-terpinene was undetectable in both free and glycosidic forms in 2018. Meanwhile, glycosidic citronellol exhibited the lowest observed concentration (2 μg/l) approaching the quantification limit in 2018.

To gain a comprehensive understanding of the genetic patterns of various monoterpenoids, the detected compounds were grouped according to their structures into three categories: chain, cyclic, and oxidized monoterpenoids. The concentrations of the compounds belonging to each group were then summed up, and the resulting data can be viewed in Tables S1 and S2. The glycosidic and total concentrations of these traits and individual compounds were subsequently analyzed as monoterpenoid traits. The concentration distribution patterns of the glycosidic form of various traits were found to be highly similar to those of the total form within the F_1_ population (Fig. S1). The most abundant compounds in the hybrids were *β*-myrcene and *β*-ocimene, which were also observed in the parent ‘Muscat of Alexandria’. The frequency histograms of the various traits showed a non-normal distribution, with a skew toward lower values. In some cases, the compounds were undetected in part of the hybrid individuals.

The majority of monoterpenoid traits, irrespective of form, exhibited a coefficient of variation >40%, with the exception of *α*-terpinene, *d*-limonene, terpinolene, and neo-allo-ocimene in 2018. The coefficient of variation of various traits ranged from 24.04% to 143.31% with regard to the total concentrations, and from 15.81% to 151.69% with regard to their glycosidic concentrations (Tables S1 and S2). Linalool exhibited the greatest variation in both forms and years.

**Figure 1:**
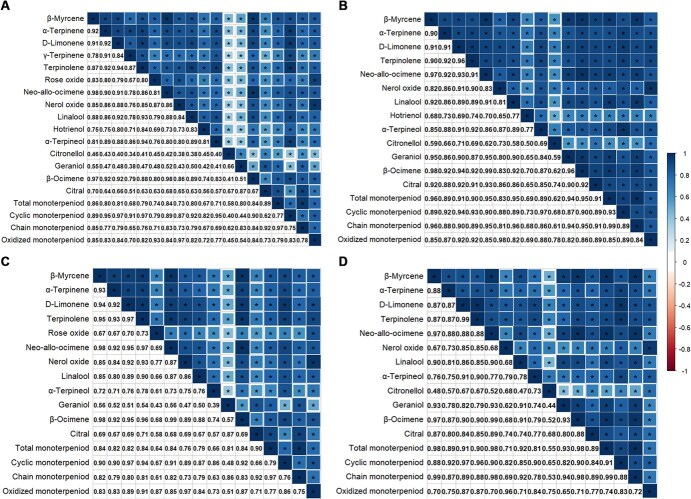
Correlation matrix of monoterpenoid traits within the F_1_ population. Correlations of total concentrations (i.e. sum of free and glycosidic forms) of various traits in 2017 (A) and in 2018 (B). Correlations of glycosidic form concentrations in 2017 (C) and in 2018 (D). The lower part of each matrix shows the Spearman correlation coefficients, which range from −1 to 1 . The ‘^*^’ in the upper part indicates a significant correlation (*P* < 0.05). The sequence of compounds corresponding to each row is the same as that of the columns, with the first substance omitted.

Given the observation that the frequency histograms of various monoterpenoid traits exhibited a similar pattern, a Spearman correlation analysis was conducted to investigate the potential relationship between these traits. Significant positive correlations (*P* < 0.05) were observed between various monoterpenoid traits in both years (Fig. 1). Most traits showed correlation coefficients >0.6, except for citronellol and geraniol, which had lower values. This suggested that the biosynthesis of these compounds might be governed by a similar regulatory mode.

### Genome-wide association analysis of monoterpenoid traits

**Figure 2:**
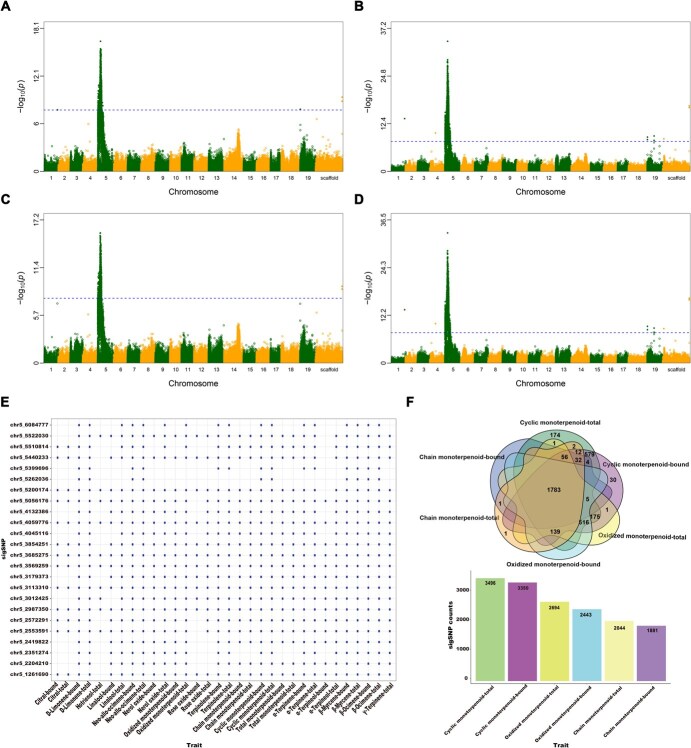
Examples of GWAS results for monoterpenoid traits. Manhattan plot of the trait ‘total monoterpenoid’ in total form in 2017 (A) and 2018 (B). Scaffold refers to chromosome 0, which means the SNP sites that are not anchored to any chromosomes in the V2.1 PN40024 grapevine reference genome. Manhattan plot of the trait ‘total monoterpenoid’ in glycosidic form in 2017 (C) and 2018 (D). The bubble matrix illustrates the correlations between 24 randomly selected significant SNPs and traits in 2017 (E). Each dot represents a significant association between the SNP and the trait, while the absence of a dot signifies the absence of an association. A Venn diagram illustrates the overlaps in number of significant SNPs associated with chain monoterpenoid, cyclic monoterpenoid, and oxidized monoterpenoid traits in both glycosidic and total forms in year 2017 (F). The bar plot shows the number of sigSNP loci associated with the abovementioned traits.

A genome-wide association study (GWAS) was conducted on the F_1_ population using 568 953 single nucleotide polymorphism (SNP) markers and a total of 68 monoterpenoid traits, including the total concentrations and glycosidic form concentrations over a 2-year period. A mixed linear model was employed for all traits. The Manhattan plots indicated that the majority of the significant SNPs (sigSNPs) were located on chromosome 5, with a considerable overlap observed among different traits (Fig. 2A–E, Figs S2 and S3). For each individual compound, the sigSNPs associated with the glycosidic and total forms exhibited a high degree of overlap, particularly those located on chromosome 5. Furthermore, this overlap was also evident among the chain monoterpenoid, cyclic monoterpenoid, and oxidized monoterpenoid traits. Figure 2F displays the number of significant SNP loci associated with the traits for total and glycosidic forms of cyclic monoterpenoids, oxidized monoterpenoids, and chain monoterpenoids, ranging from 1881 to 3496. Of the sigSNPs, 1783 loci were common to these traits. This finding is consistent with the aforementioned observation of a strong positive correlation between traits (see Fig. 1). A total of 4089 sigSNPs were identified as being associated with all traits under investigation. Of these, 4078 were located on chromosome 5, with the remaining distributed across chromosomes 1, 4, 19, and 0 (unknown chromosome). A total of 892 candidate genes were identified within the regions extending 10 kb from the various significant SNPs (Table S3). The genes were clustered densely, with 827 genes located on chromosome 5 and 36 genes located on chromosome 19.

**Figure 3:**
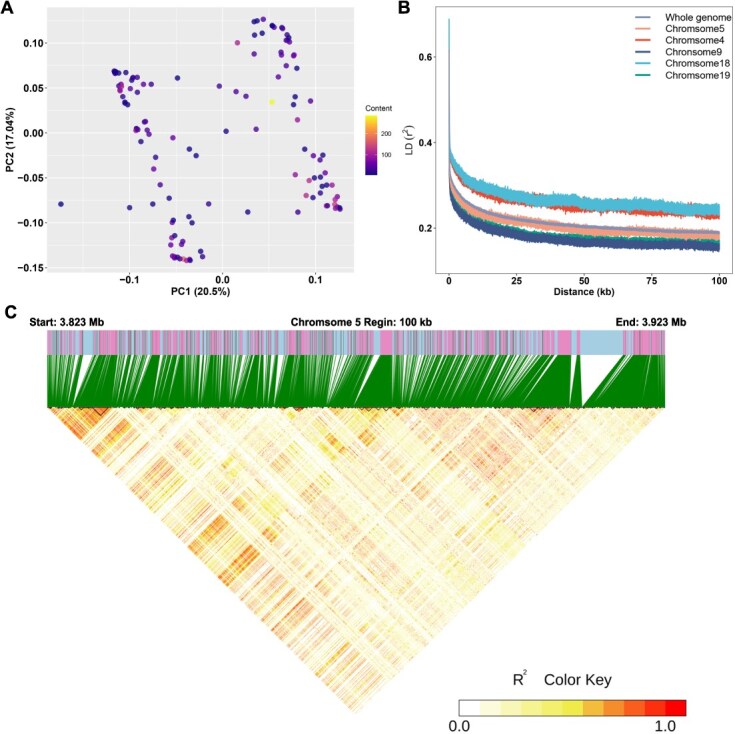
Analysis of genotype data of chromosome 5. (A) PCA plot of the genotype data of chromosome 5. Points in the plot represent F1 individuals within the hybrid population. The glycoside content of total monoterpenoid in 2017 was used as a representative of phenotype. (B) LD decay plot of chromosome 5, whole genome and other four random selected chromosomes. (C) LD heatmap of the most significant 100-kb reign of the candidate interval on chromosome 5.

To ensure that the cluster of significant SNPs spanning an 8-Mb region on chromosome 5 was genuinely associated with monoterpenoid traits rather than influenced by confounding factors such as population stratification, we first performed principal component analysis (PCA) on the genetic data of chromosome 5, including the data of parents. The results showed a random distribution of F1 individuals along the first two principal components (PC1 and PC2; Fig. 3A), with no clustering based on monoterpenoid levels, indicating the SNP cluster was not driven by population stratification. The first two PCs explained 37.54% of the variance, comparable to four other randomly selected chromosomes (Fig. S4), suggesting chromosome 5 did not exhibit exceptional genetic variability.

For a deeper insight into the genetic architecture of chromosome 5, we performed linkage disequilibrium (LD) analysis. The genome-wide profile identified a rapid LD decay within the F1 population, with chromosome 5 displaying a similar decay pattern (Fig. 3B). This suggests that there may be a high recombination rate in the hybrid population and that the detection of the 8-Mb candidate interval is unlikely to result from background LD. Compared to chromosome 5, chromosome 18 and 19 had faster LD decay, while chromosome 4 and 9 had slower decay. This improves that the recombination behavior observed on chromosome 5 reflects a genome-wide trend rather than a chromosome-specific phenomenon. Additionally, the LD heatmap across the 8-Mb interval identified 419 independent LD blocks (Fig. S5). As shown in the most significant 1-Mb subregion (Fig. 3C), multiple discrete LD blocks with limited linkage between them were found. These pieces of evidence suggest that the thousands sigSNPs on chromosome 5 were not driven by a single, strong LD spanning the entire candidate region, which supports the idea that the candidate interval contains multiple independent genetic variants contributing to the observed signal.

To validate these associations, we performed genotype–phenotype correlation analysis using a representative subset of 50 sigSNPs randomly selected from the total 4078 identified sigSNPs. Using the total monoterpenoid content in 2018 as a representative phenotype, we found significant associations between genotype and monoterpenoid levels for all selected sigSNPs (Fig. S6). Collectively, these results demonstrate that the SNP cluster on chromosome 5 is indeed associated with monoterpenoid content in the F_1_ population, confirming the robustness of our GWAS findings.

### Monoterpenoid-related marker cluster on chromosome 5

**Figure 4:**
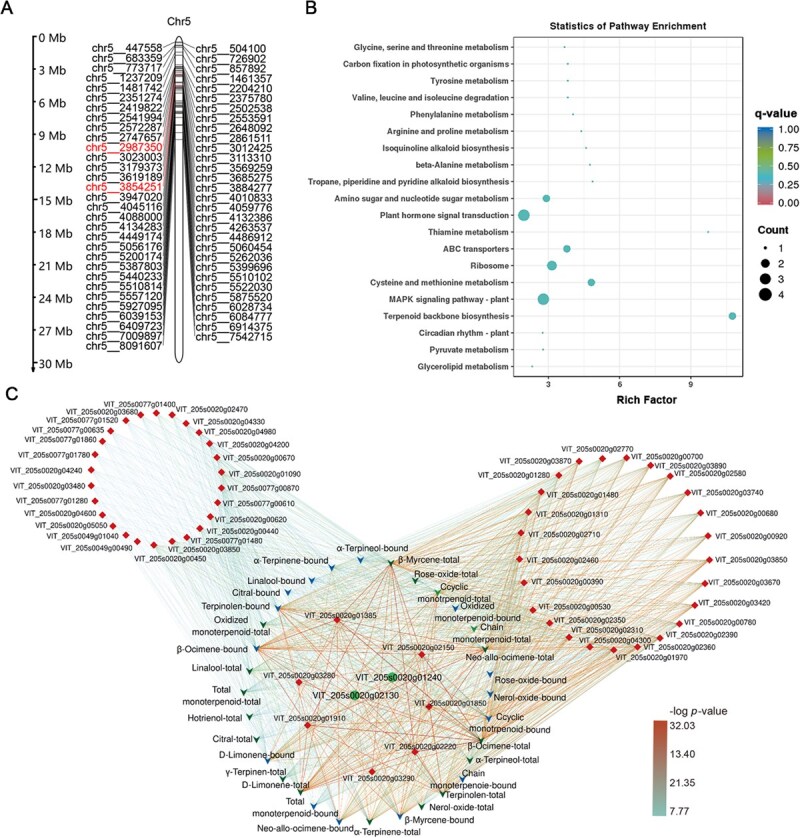
Marker SNP and gene clusters associated with monoterpenoid traits on chromosome 5. (A) Location of the SNP markers on chromosome 5. The SNPs associated with *VvDXS1* and *VvGGPPS-LSU* are highlighted. (B) The top 20 enriched KEGG pathways of the candidate marker genes. (C) Correlation network of candidate marker genes (marked as diamonds) and monoterpenoid traits (green triangle represents total form, while blue triangle represents glycosidic form). *VvDXS1* and *VvGGPPS-LSU* are highlighted in cycles. The thickness of the lines represent the *P*-values of GWAS results, with thicker lines indicating lower *P*-values.

Given that >99% of sigSNPs were concentrated on chromosome 5, we put forth a marker cluster that is associated with monoterpenoid levels. Subsequently, the lead SNPs were screened as marker SNPs based on linkage disequilibrium and *P*-value among all sigSNPs on chromosome 5. The lead SNPs were identified as the independent SNPs without LD relationships and exhibiting the lowest *P*-value located within a 10-kb window. A total of 61 stable lead SNPs were gained from the data collected over a 2-year period. All of these were located within the exon region of the candidate genes, and these lead SNPs were all concentrated in the upstream region of chromosome 5 (Fig. 4A). The 61 lead SNPs were 1:1 mapped to 61 genes. Considering the telomere-to-telomere (T2T) gap-free grapevine reference genome (PN40024 T2T) published in 2023 [35], the 61 genes were aligned with the latest version of the genome to verify the absence of pseudogenes. Results indicated that all genes mapped to chromosome 5 (Hap 1) of PN40024 T2T genome, consistent with their locations in the PN40024 V2.1 genome (Table S4).

The 61 genes were found to be enriched in the terpenoid backbone biosynthesis, hormone signal transduction, the ABC transporter pathways, and the other processes based on Kyoto Encyclopedia of Genes and Genomes (KEGG) enrichment analysis (Fig. 4B, Tables S3 and S4). The most enriched pathway was ‘terpenoid backbone biosynthesis pathway’. The two key genes involved in this pathway were found: a well-characterized 1-deoxy-D-xylulose-5-phosphate synthase 1 (*VvDXS1;* VIT_205s0020g02130) and a novel candidate geranylgeranyl pyrophosphate synthase large subunit (*VvGGPPS-LSU;* VIT_205s0020g01240). The candidate genes enriched in other pathways including three glycosidic hydrolases, three protein phosphatases, ABC-2 type transporter, two serine/threonine-protein kinases, oxygenase, transcriptional factors with AP2 /ERF domain and SBP domain, as well as some PPR repeat family members (Table S3). All candidate genes were found to be significantly associated with multiple monoterpenoid traits, with extremely low *P*-values (Fig. 4C and Table S4). This suggests that these genes may play a role in the regulation of monoterpenoid synthesis.

Specially, 233 and 187 significant SNPs were linked to *VvDXS1* and *VvGGPPS-LSU*, respectively. Two SNPs were located at chr5_3854251 (the lead SNP) and chr5_3851898, which were situated within the coding sequence (CDS) region of *VvDXS1.* These SNPs would result in a sense mutation (Table 1). The locus at chr5_3854251 has been previously characterized [26]. At this locus, a mutation from cytosine (C) to adenine (A) resulted in the substitution of lysine (K) with asparagine (N) at the 284th amino acid, thereby leading to an increase in the concentrations of the majority of monoterpenoids, which is consistent with the previous report [25]. This mutation was identified in the predominant transcript of *VvDXS1* (transcript variant 1). Another mutation at chr5_3851898, from adenine (A) to cytosine (C), occurred on the transcript variant 2 of *VvDXS1.* This resulted in the substitution of leucine (L) with arginine (R) at the 528th amino acid, which was similarly associated with an increase in monoterpenoid levels (Table 1).

**Table 1:** Information of the two marker genes enriched in terpenoid backbone biosynthesis pathway

Gene ID	Annotation	Traits 2017^a^	Traits 2018^a^	SNP loci	Position	Genotype–phenotype relationship
VIT_205s0020g02130	1-Deoxy-d-xylulose-5-phosphate synthase 1 (*VvDXS1*)	1,2,4,5,6,7,8,9,10,11,12,13,14,15,16,17^b^	1,2,3,4,5,6,7,9,10,11,12,13,14,15,16	chr5_3851898	CDS	A → C (Low→High)
				**chr5_3854251**	CDS	C → A (Low→High)
VIT_205s0020g01240	Geranylgeranyl pyrophosphate synthase large subunit (*VvGGPPS-LSU*)	1,2,4,5,6,7,8,9,10,11,12,13,14,15,16,17^b^,18^b^	1,2,3,4,5,6,7,9,10,11,12,13,14,15,16	**chr5_2987350**	CDS	C → T (Low→High)
				chr5_2988230	5′-UTR	C → G (Low→High)
				chr5_2989379	Intron	A → G (Low→High)

a The number represented glycosidic and total concentrations of different traits: 1. citral; 2. *d*-limonene; 3. geraniol; 4. linalool; 5. neo-allo-ocimene; 6. nerol oxide; 7. oxidized monoterpenoid trait; 8. rose oxide; 9. terpinolene; 10. chain monoterpenoid trait; 11. cyclic monoterpenoid trait; 12. total monoterpenoid trait; 13. *α*-terpinene; 14. *α*-terpineol; 15. *β*-myrcene; 16. *β*-ocimene; 17. *γ*-terpinene; 18. hotrienol.

b Given trait corresponds exclusively to its total concentration (sum of free and glycosidic forms). It should be noted that neither *γ*-terpinene nor rose oxide was detected in the grapes of 2018. The lead SNPs are presented in boldface. CDS represents the coding sequence, while 5′-UTR represents the 5′-untranslated region.

Three SNP loci were identified within the gene region of *VvGGPPS-LSU.* These were located at chr5_2987350 of the CDS region (being the lead SNP), chr5_2988230 of the 5′-untranslated region (5′-UTR), and chr5_2989379 of the intron region. The loci were found to be significantly associated with the glycosylated forms and total contents of other 11 monoterpenoid compounds in both years, except for geraniol (No.3), oxide rose (No.8), *γ*-terpinene (No.17), and hotrienol (No.18). Among these four compounds, neither *γ*-terpinene nor rose oxide was detected in the 2018 grape samples, while geraniol and hotrienol were not significantly associated in 2017 and/or 2018 (Table 1). The mutation from cytosine (C) to thymine (T) at chr5_2987350 would result in an amino acid substitution at position 261 of VvGGPPS-LSU, from glycine (G) to arginine (R), and higher concentrations of multiple monoterpenoid traits within the F_1_ population (Table 1).

### Verification of candidate SNP markers in germplasm population

The reliability of the 61 candidate SNP markers was validated in a grapevine germplasm population comprising 97 Vitis species using kompetitive allele-specific PCR (KASP). The genotypes of 53 candidate markers were examined (Table S5), while the remaining eight SNPs were not considered due to the unavailability of suitable primers (Table S6). Furthermore, the concentrations of various monoterpenoid compounds were quantified in the berries of these grapevine varieties. β-ocimene, trans-β-ocimene, and linalool displayed the highest concentrations in this germplasm population, which was similar to the situation observed in the hybrid population. The concentration of individual compounds exhibited considerable variation among the 97 species, with a range of 5- to 500-fold (Table S7). β-citral demonstrated a particularly notable variation, reaching 558-fold. Additionally, β-ocimene, linalool, terpinen-4-ol, linalool oxide, and nerol oxide exhibited concentrations that varied by 100-fold. Moreover, a positive correlation was identified between the concentration of individual compounds and the total monoterpenoid content (*P* < 0.05) (Fig. S7). It was proposed that the total monoterpenoid could be used as a phenotypic representation to elucidate the impact of diverse genotypes at SNP loci.

**Figure 5:**
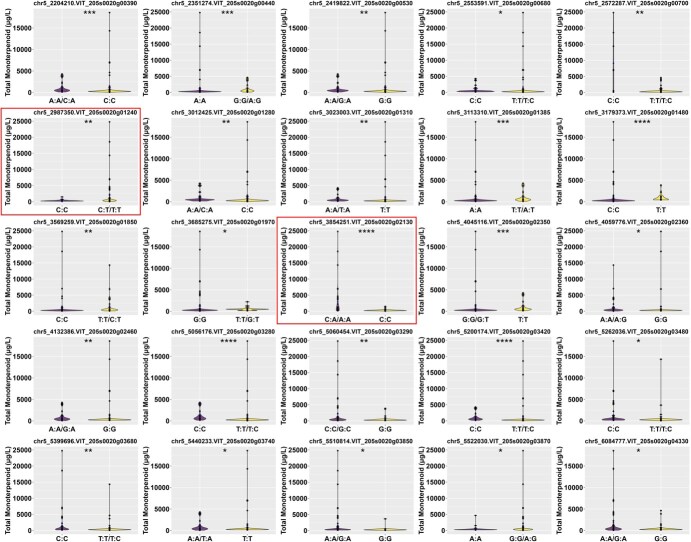
Concentration distribution violin plots of different genotypes at lead SNP loci in the germplasm population. chr5_2987350 located on *VvGGPPS-LSU* and chr5_3854251 located on *VvDXS1* were highlighted in boxes. The total concentration of monoterpenoids (μg/l) in grape berries was used as phenotypic representation. Asterisks denote Kruskal test significance: ^*^P < 0.05; ^**^P < 0.01; ^***^P < 0.001; ^****^P < 0.0001.

Of the 53 lead SNPs that were tested, the different alleles of 32 of these were subsequently proved to be associated with monoterpenoid levels. It was noteworthy that 25 of these loci demonstrated observable variation in monoterpenoid concentration with respect to the allele, including chr5_2204210, chr5_2351274, chr5_2419822, chr5_2553591, chr5_2572287, chr5_2987350, chr5_3012425, chr5_3023003, chr5_3113310, chr5_3179373, chr5_3569259, chr5_3685275, chr5_3854251, chr5_4045116, chr5_4059776, chr5_4132386, chr5_5056176, chr5_5060454, chr5_5200174, chr5_5262036, chr5_5399696, chr5_5440233, chr5_5510814, chr5_5522030, and chr5_6084777 (Fig. 5). These SNPs can be regarded as reliable markers for further investigation. *Vitis* species with the C/T or T/T genotype at the chr5_2987350 locus (*VvGGPPS-LSU*) showed higher monoterpenoid levels than those with the C/C genotype. For the chr5_3854251 locus on *VvDXS1*, the homozygous C/C genotype was associated with lower monoterpenoid levels compared to the A/A or C/A genotypes. These findings align with the F1 population data, supporting the reliability of the 25 lead SNP loci as markers for monoterpenoid levels.

**Figure 6:**
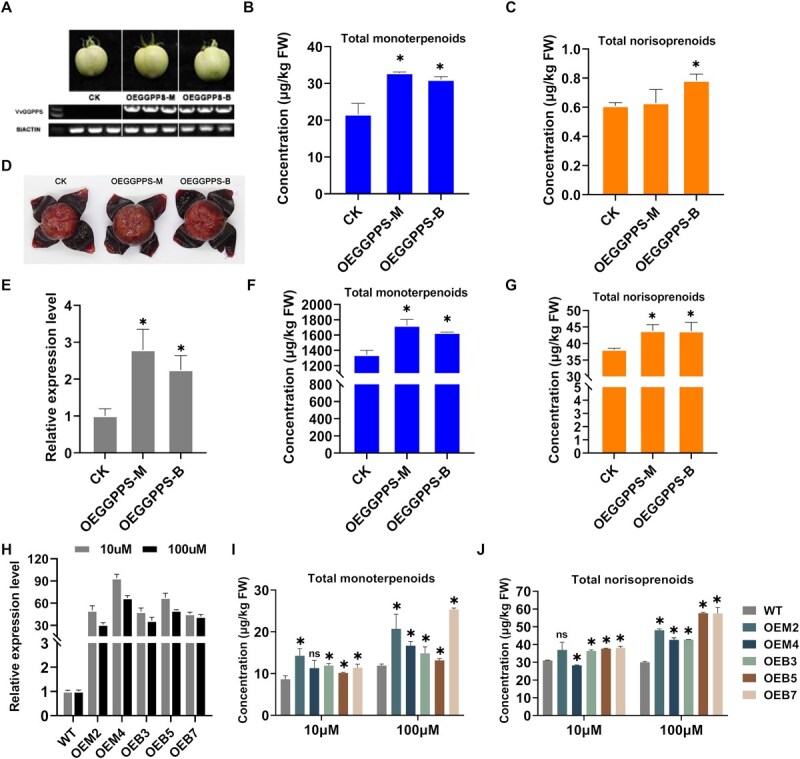
Effects of *VvGGPPS-LSU* overexpression on the concentrations of monoterpenoids and norisoprenoids in tomato fruits, grape skins, and grape calli. (A) Representative images and RT-PCR analysis of transiently overexpressed Micro-Tom tomato fruits. (B) Total concentrations of monoterpenoids and (C) norisoprenoids in the control with an empty vector (CK) and transiently overexpressed tomato fruits. OEGGPPS-M and OEGGPPS-B represent the *VvGGPPS-LSU* gene from ‘Manseng Petit Blanc’ and ‘Muscat Blanc 455’, respectively. (D) Image of the ‘Summer Black’ grapes employed for transient overexpression. The analysis was conducted exclusively on the skins. (E) Relative expression levels of *VvGGPPS-LSU*, total concentrations of (F) monoterpenoids and (G) norisoprenoids in grape skins of the control (CK), OEGGPPS-M and OEGGPPS-B. (H) Relative expression levels of WT *V. vinifera* callus and stable overpassed lines of VvGGPPS-LSU from ‘Manseng Petit Blanc’ (OEM2, OEM4) and ‘Muscat Blanc 455’ (OEB3, OEB5, OEB7) added 10 or 100 μM mixed IPP and DMAPP. Data are present as means ± SD of three biological replicates. (I) Total concentrations of monoterpenoids and (J) norisoprenoids in the WT *V. vinifera* calli and stably overexpressed lines with the application of 10 or 100 μM IPP and DMAPP. All bar plot data are presented as means ± SD of at least three biological replicates. The statistical significance of the data was calculated using an unpaired *T-test (P* < 0.05; ns means not significant difference).

### Functional validation of *VvGGPPS-LSU* in monoterpenoid accumulation

To investigate the function of the novel candidate *VvGGPPS-LSU* in regulating monoterpenoid synthesis, the coding sequences of the genes were cloned from *Vitis vinifera* L. ‘Muscat Blanc 455’ (B) and *V. vinifera* L. ‘Manseng Petit Blanc’ (M) for further analysis. A preliminary study revealed that the chr5_2987350 locus was a T/T allele in the *VvGGPPS-LSU* of ‘Muscat Blanc 455’ (abbreviated as *VvGGPPS*-B) and a C/C genotype in the *VvGGPPS-LSU* of ‘Manseng Petit Blanc’ (*VvGGPPS*-M) (Fig. S8). Both *VvGGPPS*-B and *VvGGPPS*-M contain two conserved aspartate-rich motifs (FARM and SARM) (Fig. S9), which are crucial for substrate binding [36]. The subcellular localization of VvGGPPS-LSU was investigated in tobacco leaves using a pHB-EGFP vector and a laser confocal microscope. *VvGGPPS*-B was used as representative, the results demonstrated that the protein localizes to the plastid, which is consistent with its expected functionality (Fig. S10).

Subsequently, gene overexpression was conducted to assess the impact of this manipulation on monoterpenoid levels. In this study, the norisoprenoid levels were also of interest, given that our previous research has demonstrated that the VvGGPPS-LSU can enhance norisoprenoid levels in tobacco and grape leaves with transient overexpression [37]. In Micro-Tom tomato fruits transiently overexpressing *VvGGPPS*-B and *VvGGPPS*-M, respectively, a total of 16 monoterpenoid components were identified, with chain monoterpenoids being predominant, as observed in grape berries. The overexpression of *VvGGPPS-LSU* resulted in a significant increase in the levels of 15 monoterpenoid compounds, with the exception of *β*-ocimene (Table S8). Consequently, the total concentrations of all compounds were observed to be elevated in the fruits overexpressing *VvGGPPS*-B and *VvGGPPS*-M, respectively, in comparison to those overexpressing the empty vector (CK) (Fig. 6B). Furthermore, four norisoprenoid compounds were identified, with their total concentrations increased by 1.27- and 1.03-fold, respectively. The overexpression of *VvGGPPS*-B significantly increased norisoprenoid in comparison to the control, with considerable elevation of 160.2% in *β*-damascenone (Fig. 6C, Table S8).

Additionally, overexpression experiments were conducted on the ‘Summer Black’ (*V. vinifera* × *Vitis labrusca*) grapes (Fig. 6D, Fig. S11). The real-time quantitative polymerase chain reaction (RT-qPCR) analysis demonstrated the successful overexpression of *VvGGPPS*-M and *VvGGPPS*-B in grape skin, with a 2.78- and 2.25-fold increase, respectively (Fig. 6E). The grape skin was found to contain a greater diversity of monoterpenoid compounds than those of norisoprenoids. Of the 20 monoterpenoid compounds identified in the skin, citronellol displayed the highest levels, followed by rose oxide, *d*-limonene, *α*-terpinene, and *β*-ocimene (Table S9). In comparison to the control sample containing the empty vector, the overexpression of *VvGGPPS*-M resulted in a 29% increase in the total concentration of monoterpenoids and a 15% increase in norisoprenoids. Similarly, overexpression of *VvGGPPS*-B led to a 22% elevation in the total concentration of monoterpenoids and a 15% increase in norisoprenoids (Fig. 6F and G). As observed in tomato fruits, overexpression of *VvGGPPS-B* or *VvGGPPS*-M resulted in a notable elevation in the levels of 17 out of 20 monoterpenoid compounds. Additionally, overexpression of *VvGGPPS*-M caused a significant increase in the levels of two out of the six norisoprenoid compounds, *β*-damascenone and *cis*-theaspirane (Table S9).

The observation that overexpressing *VvGGPPS-LSU* enhanced both monoterpenoids and norisoprenoids leads to the supposition that VvGGPPS-LSU possesses dual functionality as geranyl diphosphate synthase (GPPS) and geranylgeranyl diphosphate synthase (GGPPS). To verify this supposition, the compounds in grape calli with *VvGGPPS-LSU* stably overexpressed were examined. A mixture of two substrates, isopentenyl diphosphate (IPP) and dimethylallyl diphosphate (DMAPP), was applied at a concentration of 10 μM or 100 μM to the surface of the overexpressed calli and wild-type (WT) calli, respectively, with the objective of supplementing the concentrations of substrates. All stable overexpression lines exhibited a 31- to 67-fold increase in relative expression levels of *VvGGPPS-LSU* in comparison to WT (Fig. 6H). Meanwhile, the addition of 10 μM mixed substrates resulted in an elevation in monoterpenoid and norisoprenoid concentrations in two *VvGGPPS*-M overexpression lines (OEM2 and OEM4) and three *VvGGPPS*-B overexpression lines (OEB3, OEB5, and OEB7), with the exception of the monoterpenoids of OEM4 and the norisoprenoids of OEM2 (Fig. 6I and J, Table S10). In the WT calli, monoterpenoids and norisoprenoids did not exhibit a significant increase in the concentration when the substrate mixture was applied at concentrations ranging from 10 to 100 μM concentration. However, each overexpressed line exhibited elevated levels of both monoterpenoids and norisoprenoids with the introduction of higher concentrations of IPP and DMAPP (Table S10). The aforementioned results demonstrate that VvGGPPS-LSU not only performs its predicted function, namely the synthesis of GGPP and thus the provision of the precursor for the biosynthesis of norisoprenoids, but also functions as GPPS, synthesizing GPP and then converting it into monoterpenoids under the catalysis of monoterpenoid synthase.

## Discussion

QTL mapping is a common and effective approach for studying quantitative traits in hybrid populations and has been widely used in many fruit species to elucidate the genetic basis of commercially important traits such as crop yield, soluble solids concentration, firmness, and flavor traits [38–41]. Since 2006, multiple QTL mapping studies focusing on monoterpenoids have been carried out in grapevine research. However, aside from *VvDXS1* [24, 42], *VvCYP76F14* [17], and *VvbZIP61* [43], no other candidate genes or practical markers have been conclusively identified. Thus, it is imperative to explore alternative approaches with higher resolution and efficiency.

To identify novel candidate genes and breeding markers associated with monoterpenoid levels, we implemented GWAS in an F_1_ hybrid population for the following reasons. First, highly heterozygous nature of the grapevine genome means that an F_1_ hybrid population can provide substantial genetic diversity, which is crucial for an effective association analysis. Second, the relatively simple population structure of an F_1_ hybrid population reduces the risk of spurious associations, thereby enhancing the statistical power to detect true trait–marker relationships. Finally, compared to QTL mapping, GWAS offers higher resolution, enabling the identification of genetic associations at the gene level, rather than broader chromosomal regions. The higher resolution is advantageous for pinpointing SNP markers and candidate genes related to traits. In this study, using GWAS, we identified a candidate gene cluster associated with multiple monoterpenoid traits in an 8-Mb interval on chromosome 5. Within the interval, there were 61 genes that are 1:1 associated with 61 independent and stable lead SNPs.

### Prevalence of gene clusters regulating specific metabolites in plants

In this study, candidate genes associated with monoterpenoids were clustered on chromosome 5. Multiple studies have shown a strong association between chromosome 5 and grape’s muscat aroma [24, 42, 44, 45]. Our results further reinforce its importance in flavor-related traits including the glycosidic form monoterpenoid. Previous studies often used the free content of few monoterpenols (such as linalool, nerol, *α*-terpineol) as phenotypes or treated aroma as qualitative trait. Including *VvDXS1*, the candidate genes on chromosome 5 found in these studies were all located in the 8-Mb interval found in our study. Twelve of the 61 important candidate genes we found were overlapped with the candidate genes reported by Lin *et al.* [44], further demonstrating the reliability of the gene cluster we identified. In addition, our study extends more candidate genes on chromosome 5. While flavor loci have also been reported in other genome regions, such as *VvbZIP61* on chromosome 12 ^44^ and two genes on chromosome 18 identified through GWAS with structural variations (SVs) integrated [46], these genes did not overlap with those found in our study, indicating the diversity of flavor loci.

The genetic mechanism of quantitative traits is generally complex and often involves the cumulative effects of multiple genes. Major QTLs containing one or few genes can significantly affect traits. In grapevines, the muscat flavor trait is primarily controlled by a major QTL linked to the *VvDXS1* gene. A single nucleotide polymorphism (SNP 1822) in the *VvDXS1* gene results in a substitution of lysine with asparagine at position 284 of the protein, which significantly increases monoterpenoid levels and affects the muscat flavor [25]. Similarly, a cluster of four MYB transcription factors has been identified as the major QTL for anthocyanin regulation in grapes [47]. In tomato, the *FW2.2* gene, a member of the cell number regulator family, has been identified as the major QTL controlling fruit weight [48].

Our present study revealed a cluster comprising 61 genes, in which the *VvDXS1* and *VvGGPPS-LUS* genes were included. Unlike major QTLs, trait-related gene clusters consist of more genes with different functions, reflecting the combined effects of major genes and multiple minor genes. The phenomenon of multiple genes forming a cluster to jointly control a trait has been reported in some plants. For example, in tomato, a gene cluster linked to monoterpenoid synthesis on chromosome 8 includes six *SlTPS* genes, two *cis*-prenyltransferase genes (*SlNDPS1* and *SlCPT8B)*, and two *SlCYP71* genes [49, 50]. Additionally, a gene cluster controlling momilactone (a diterpenoid compound) levels has been found in rice [51]. Researchers suggest that the clustering of genes controlling the same trait is of great biological significance for metabolic pathway coordination. These gene clusters typically encode enzymes involved in specific metabolic pathways, enabling the efficient synthesis of secondary metabolites crucial for plants. The coordinated expression and regulation of these genes allow plants to rapidly respond to environmental stresses by producing defensive compounds [52, 53]. This genomic organization is a result of evolutionary pressures and provides insights into the genetic basis of plant metabolic diversity and specialization [54, 55].

### Potential function of the gene cluster in regulating monoterpenoids

In this study, the candidate genes clustered on chromosome 5 were enriched in various metabolic pathways. These gene included not only *VvDXS1* and *VvGGPPS-LSU* in terpenoid biosynthetic pathway, but also ABC transporter, protein phosphatase 2C (PP2C) family members, serine/threonine-protein kinases, and transcription factor SBP, among others. These genes may be involved in the transformation and transportation of monoterpenoids as well as the transcriptional regulation of these processes in grapes. ABC transporters has been demonstrated to be involved in terpenoid transport in flowers and other plants [56, 57]. For example, two ABC transporters, PbABCG1 and PbABCG2, have been shown to participate in the emission of monoterpenoids in *Phalaenopsis bellina*, thereby altering the balance between internal monoterpene pools and emitted pools [58]. Additionally, SmABCG1 has been shown to facilitate the export of tanshinone (a diterpenoid) from peridermic cells, indirectly regulating its biosynthesis [59].

Protein phosphatases and protein kinases frequently participate in defensive processes and phytohormone signaling pathways in plants [60, 61]. Currently, there is no evidence suggesting a direct influence of these proteins on monoterpenoid metabolism. However, as secondary metabolites, monoterpenoids play a crucial role in plant resistance to biotic and abiotic stresses [62, 63]. Many studies have demonstrated that monoterpenoids play a crucial role in plant resistance through various signaling pathways, often involving phytohormones such as jasmonic acid [64–66]. Based on the aforementioned information, it can be posited that protein phosphatases and protein kinases may regulate monoterpenoid metabolism through a complex mechanism that requires further exploration.

Research on biosynthetic gene clusters in plants has primarily focused on structural genes due to limited understanding of transcriptional regulation. However, emerging studies suggest that transcription factors may exert global regulatory effects on gene clusters. For example, OsTGAP1, located on the same chromosome as the momilactone biosynthesis gene cluster in rice, has been shown to be essential for momilactone biosynthesis and is involved in the transcriptional regulation of five genes within the cluster [67]. In melon, the bHLH transcription factor CmBr has been demonstrated to directly activate the expression of eight structural genes involved in the biosynthetic pathway of cucurbitacin B, the major bitter triterpenoid compound in melon [68].

The SBP transcription factor identified in our study may function similarly. Also known as SQUAMOSA Promoter-Binding Protein-Like (SPL), these factors have been shown to regulate terpenoid levels in lavender, *Artemisia annua*, *Arabidopsis thaliana*, Patchouli, and tea through an interaction with miR156, which in turn activates terpene synthases [69–73]. Specifically, LiSBP-2 has been demonstrated to activate the expression of linalool and 1,8-cineole synthases in lavender [69], highlighting the potential regulatory roles of SBP family members in monoterpenoid biosynthesis.

The confirmed relationship between SNP clusters and monoterpenoid content in the germplasm population suggests that this gene cluster may co-regulate monoterpenoid metabolism. However, comprehensive functional validation of these candidate genes is still required, such as through transgenic or gene-editing experiments, to confirm their roles in grapevine monoterpenoid metabolism. Such studies will not only elucidate the regulatory mechanisms of grape flavor but may also uncover novel insights into plant monoterpenoid metabolism.

### Role of VvGGPPS-LSU in isoprenoid-derived aroma metabolism

GGPPS serves as a pivotal branch point enzyme in isoprenoid biosynthesis, regulating the conversion of geranyl diphosphate (GPP) to geranylgeranyl diphosphate (GGPP). GPP is the direct precursor for monoterpenoids, while GGPP indirectly contributes to norisoprenoid biosynthesis via carotenoid cleavage [74–76]. Thus, VvGGPPS-LSU activity may significantly influence the levels of both monoterpenoid and norisoprenoid aromas. Our previous work highlighted its role in norisoprenoid production [37], and the current study demonstrates that overexpressing VvGGPPS-LSU elevates both monoterpenoid and norisoprenoid levels in grapes.

In plants, GGPPS comprises both the large subunit (LSU) and the small subunit (SSU). The LSU is typically regarded as the functional unit, while the SSU is generally considered a noncatalytic unit that can interact with the LSU to form a functional heterodimer [77]. In eucalyptus, *GGPPS* expression has been shown to be significantly correlated with *p*-cymene levels and the ratio of monoterpenoids to sesquiterpenoids in leaves [78, 79]. A study on *Osmanthus fragrans* revealed that the heterogenic OfGPPS is formed by two LSUs interacting with SSUII, thereby enhancing monoterpenoid biosynthesis [80]. These findings support the hypothesis that GGPPS-LSU can perform functions analogous to those of GPPS.

The grapevine GGPPS family includes six members: one VvGPPS, two VvGGPPSs, two VvGGPPS-SSUs, and one VvGGPPS-LSU (VIT_205s0020g01240). Among these, *VvGGPPS1* (VIT_204s0023g01210) is linked to carotenoid production [81], while *VvGGPS-SSU1* (VIT_219s0090g00530) correlates with monoterpenoid accumulation during grape ripening, as evidenced by RNA-seq data [82]. Previous studies indicate that LSU–SSU interactions can influence metabolic flux toward GGPP or GPP synthesis [83–85], though further research is needed to clarify this relationship in grapevines.

This study confirms the dual role of VvGGPPS-LSU in controlling monoterpenoid and norisoprenoid biosynthesis. We propose that substrate availability (IPP and DMAPP) determines whether GGPPS-LSU generates GPP or GGPP, thereby affecting the production of these aroma compounds. By manipulating IPP and DMAPP levels in overexpressed grape calli, we validated VvGGPPS-LSU's dual functionality as both GPPS and GGPPS. These insights enhance our understanding of aroma biosynthesis regulation and offer strategies for targeted molecular breeding to improve grape aroma profiles.

### Development of marker set targeting monoterpenoids in grape breeding

Molecular marker-assisted selection (MAS) is a breeding technology that accelerates processing times and reduces management costs compared to conventional methods. It is less affected by environmental factors and allows for early detection of traits during plant growth. Identifying markers linked to specific traits is crucial for the success of MAS. GWAS are effective for selecting potential MAS markers. Currently, the chr5_3854251 (K284N) SNP in VvDXS1 is the only identified marker associated with monoterpenoid levels [25]. However, this SNP is absent in some aromatic grape varieties [26], indicating the need to identify and validate additional genetic markers.

GWAS is an effective approach for selecting potential markers for MAS. In this study, GWAS revealed numerous novel SNP loci associated with monoterpenoid variation, of which 25 were validated as robust markers across a diverse *Vitis* germplasm panel (Fig. S12). The variation in monoterpenoid levels was attributed to differing allele combinations in the 25 SNP markers, including the reported chr5_3854251 on *VvDXS1*. To facilitate marker detection and streamline early selection in breeding programs, the SNP marker set was converted to a KASP marker set, with primers provided. KASP technology was selected for its high specificity in allele discrimination and capacity for multiplexing, making it ideal for large-scale breeding applications. To further improve cost-effectiveness, future work will optimize electrophoresis-based markers such as CAPs (Cleaved Amplified Polymorphic Sequences) and dCAPs (Derived CAPs), ensuring broader accessibility for resource-limited breeding programs targeting monoterpenoid-related traits.

This study examined the link between SNP markers, candidate genes, and grape monoterpenoid levels, while the other genomic variants were not considered. Recent grape pangenome research have highlighted the substantial impact of SVs on traits like disease resistance and observable traits (e.g. fruit color, seedless) [46, 86]. As Long *et al.* [87] noted, SVs frequently occur in aroma-related genes between wild and domesticated populations. Hence, future research should use grape pan-genome data to comprehensively analyze SVs tied to monoterpenoid metabolism. Also, our GWAS found hundreds of candidate genes associated with monoterpenoid biosynthesis. Identifying key genes and developing functional molecular markers from these candidates is critical. Recent studies on grape seedlessness and pest resistance have shown that combining comparative genomics, transcriptomics, and artificial intelligence approaches like deep learning works well [88, 89]. These integrated approaches have clarified these traits’ genetic basis and created effective predictive models, providing new ways to understand GWAS results and applying them in breeding. Applying such multiomics and AI-integrated analytical frameworks to our study can better unravel grape monoterpenoid biosynthesis genetic regulatory network. This would allow precise monoterpenoid content prediction and targeted selection of breeding materials with desired traits.

## Materials and methods

### Plant materials

The F_1_ population was derived from a cross between two distinct varieties of table grapes. The paternal parent *V. vinifera* L. Muscat of Alexandria is an ancient Muscat cultivar with a high monoterpenoid level, while the maternal parent is *V. vinifera* L. Christmas Rose, a non-aromatic (also called as neutral-type) variety with a low monoterpenoid level. Detailed information regarding this population and authentic hybrid identification is available in our recent publication [37]. The grapes were harvested in 2017 and 2018 when the total soluble solids (TSS) reached ~16°Brix. More than 100 g berries were collected for each individual, transported to the laboratory, and frozen in liquid nitrogen. Subsequently, the samples were stored at −80°C for future analysis.

A germplasm population containing 97 grapevine varieties was sourced from the Fruit Research Institute, Shanxi Academy of Agricultural Sciences (37°42′N, 112°55′E). In 2019, young leaves and ripening berries of all varieties were collected with the objective of validating significant SNP loci obtained through GWAS using KASP.

Tomato (*Solanum lycopersicum* cv MicroTom) and tobacco (*Nicotiana benthamiana*) plants were cultivated in a growth chamber with a 16-h light and 8-h dark cycle at a temperature of 25 ± 1°C. *Vitis vinifera* L. Cabernet Sauvignon grape non-embryonic calli were cultured on B5 medium in the dark at 25°C, following the protocol established by Meng *et al*. [90]. Mature ‘Summer Black’ grapes were obtained from a commercial vineyard.

### Measurement of monoterpenoids

The aroma compounds of grape berries were extracted using the method previously described [91]. Berries were ground into a powder under the protection of liquid nitrogen. The samples were then thawed at 4°C for 4 h and centrifuged at 8000 rpm for 15 min. The resulting supernatant was used to determine free-form aroma compounds, with 5 ml being utilized for analysis.

To isolate glycosidic-bound precursors, we added 2 ml of the supernatant to a Cleanert PEP-SEP cartridge (PE1506; Bonna-Agela Technologies, USA) that has been preconditioned with 10 ml of methanol and 10 ml of Milli-Q water. The mixture was eluted with 2 ml of water and 5 ml of dichloromethane, and the glycosidic fraction was obtained by eluting it with methanol. The methanol was then evaporated, and the residue was redissolved in a 10 ml citrate–phosphate buffer solution (pH = 5.0). The aromatic aglycone was released through enzymatic hydrolysis using commercial glycosidase AR2000 (DMS Food Specialties Beverages Ingredients, Delft, Netherlands). For gas chromatography and mass spectrometry (GC–MS) analysis, 5 ml of the supernatant or enzymatic hydrolysate, along with 10 μl of 4-methyl-2-pentanol (as an internal standard, 1.024 g/l).

The aroma compounds of grape calli were measured using a published methodology [92]. For the determination of tomato aroma compounds, a sample powder weighting 4 g was blended with 10 μl of 4-methyl-2-pentanol internal standard (0.1024 g/l) and 2 g of CaCl_2_ in a vial prior to detection.

Aroma compounds were detected using handspace solid-phase microextraction-gas chromatography and mass spectrometry (HS-SPME-GC–MS), following the methodology from our previous study [90, 92]. GC–MS analysis was conducted using an Agilent 6890 gas chromatograph coupled with an Agilent 5975C mass spectrometer. An HP-INNOWAX capillary column was used to separate compounds. Monoterpenoids were identified using standards or retention indexes and quantitatively evaluated through standard calibration curves, following the published method [91].

### Genome-wide association study

The parents and 126 hybrids were sequenced using Illumina HiSeq technology and mapped to version 12X.v2 of the PN40024 grapevine reference genome with v2.1 annotation (https://urgi.versailles.inra.fr/Species/Vitis). SNP calling and GWAS model were described by Sun *et al.* [37]. The traits measured were the concentrations of glycosidic-form and total concentrations (the sum of free-form and glycosidic-form of each individual) of 15 monoterpenoid compounds over a period of 2 years. Additionally, the total concentration of all monoterpenoid compounds (referred to as total monoterpenoids) and the sum of cyclic, chain, and oxidized monoterpenoid sets per individual were also computed as traits. The significant threshold of SNPs associated with each trait was calculated by 1000 permutation tests (*P* < 0.05). The candidate genes consisted of the genes found within a 10-kb radius of sigSNPs. The proteins encoded by these genes were annotated using the Swissprot protein database (https://www.expasy.org/resources/uniprotkb-swiss-prot), and the Non-Redundant Protein Sequence Database (https://www.ncbi.nlm.nih.gov/refseq/about/nonredundantproteins/).

### Candidate SNP marker verification

Lead SNPs are defined as the sigSNPs exhibiting the strongest association with a particular trait within a genomic region, and displaying high LD with other SNPs. Lead SNPs of year 2017 and 2018 were calculated in PLINK v1.90 with parameters –clump-p1 0.000001 –clump –p2 0.01 –clump-kb 10 –clump-r2 0.50. GWAS association results of different traits were combined as input. The lead SNPs located within gene regions and stable in 2 years were considered as candidate markers of monoterpenoids, and further validated using KASP in the germplasm population. DNA of all *Vitis* species were extracted using Bioteke Rapid Plant Genomic DNA Extraction Kit. Each lead SNP has two allele-specific forward primers and one common reverse primer as listed in Table S6. The PCR fragments containing the alleles of each SNP were amplified using KASP-TF Master Mix (LGC Genomics, UK) and analyzed with the GeneMatrix high-throughput genotyping system.

### Gene overexpression in tomato, grape skin, and grape calli

The coding sequence of *VvGGPPS-LSU* was amplified from ‘Muscat Blanc 455’ (B) and ‘Manseng Petit Blanc’ (M) grapes, respectively. The primers were designed based on the sequence information from 12X.2 version of the grapevine reference genome (https://urgi.versailles.inra.fr/Species/Vitis) as listed in Table S11. PCR fragments were verified by agarose gel electrophoresis and sequencing. Subsequently, they were introduced into separate expression vectors, pCAMBIA1300, pHB-EGFP, and pCXSN. These were sequenced again to ensure accuracy. The resulting recombinant fragments were designated as ‘*VvGGPPS*-B’ and ‘*VvGGPPS*-M’, respectively.

For the transient overexpression in tomato fruits, the pCAMBIA1300-recombined plasmids were transferred to the strain EHA105 of *Agrobacterium tumefaciens*. The transformation method was performed as detailed by Hoshikawa *et al.* [93] with some modifications. Initially, EHA105 harboring the pCAMBIA1300 vector was precultured overnight in Luria–Bertani (LB) medium with 50 mg/l of kanamycin and 20 mg/l of rifampicin at 28°C. The following day, 1 ml of the culture was transferred to a fresh 50 ml of the same medium. On the third day, the culture was centrifuged and suspended in the infiltration buffer (10 mM MES, 10 mM MgCl_2_⋅6H_2_O, 200 μM acetosyringone, pH = 5.6). The solution was injected into tomato fruit 3 days before breaker stage. Fruits were collected 3 days after injection.

The pHB-EGFP recombined plasmids, with *VvGGPPS*-B and *VvGGPPS*-M fused to the N-terminal of EGFP, respectively, were transferred to the strain GV3101 for the transient overexpression in grape skin. The *Agrobacterium* cells were resuspended in the buffer reported by Mu *et al.* [94]. About 15 berries from three clusters of mature ‘Summer Black’ were used as three biological replicates. Berries were washed and pierced with 20 holes to reduce internal pressure. They were then immersed in 200 ml of *Agrobacterium* suspension and vacuum (0.09 MPa) infiltrated for 15 min. After slowly returning to atmospheric pressure, the berries were dried and cultured in the growth chamber with a 16-h light/ 8-h dark cycle at 26°C. Skins were collected after 3 days.

The grape calli were transformed stably following the protocol published by Meng *et al.* [90]. The pCXSN vector recombined plasmids were introduced into the strain GV3101, while the WT calli were used as a control. The calli were located on B_5_ medium containing Hygromycin B to select and maintain transgenic lines, and transferred to fresh medium every 4 weeks. Three days before collection, the calli were incubated with 10 and 100 μM IPP and DMAPP, which are substrates for GPP, the universal precursor of monoterpenoids. The objective was to improve the levels of monoterpenoids.

### RNA extraction, RT-PCR, and RT-qPCR

To test if the genes were successfully overexpressed in tomato fruits, grape skins, and grape calli lines, total RNA was extracted using the Universal Plant Total RNA Extraction Kit (Bioteke, China). The primers used were shown in Table S11. For tomato fruits, the extracted RNA was used to synthesize cDNA utilizing commercial kit (R212, Vazyme, China). The PCR procedure was carried out according to the manufacturer’s instructions and performed in a C1000 Touch™ Thermal Cycler (Bio-Rad, USA). SlACTIN was used as the reference gene.

To analyze gene expression in grape calli lines and grape skins, we performed the reverse transcription of RNA to cDNA using kit from Vazyme (R223, China). RT-qPCR analysis was then conducted on a Bio-Rad CFX96TM system (Bio-Rad, USA) using the SYBR qPCR Master Mix (Q711, Vazyme, China), with the VvUBIQUITIN gene serving as reference. All corresponding primer sequences were listed in Table S11. The gene expression levels were calculated by 2^-ΔΔCT^ method in CFX Maestro Software.

### Subcellular localization

For subcellular localization, the pHB-EGFP *Agrobacterium* cells were injected into 4-week old *N. benthamiana* leaves as reported previously [37] and cultured in a growth chamber for 3 days. The signal of leaves were detected using a ZEISS LSM710 confocal system (ZEISS, Germany) with an excitation wavelength at 488 nm for the EGFP fluorescent and 543 nm for chlorophyll autofluorescence.

### Statistical analysis

The histogram was generated using the R package ‘ggplot2’. The Spearman correlation coefficient was computed by applying the ‘cor’ function. Furthermore, the heatmap was constructed using R package ‘corrplot’, ‘ggpubr’ in version 4.1.3 of R. Bar plots were generated using GraphPad Prism 9 for Windows (MA, USA) and statistical significance was assessed by performing an unpaired *t-*test (significant levels: ^*^P < 0.05, ^**^P < 0.01, ^***^P < 0.001, ^****^P < 0.0001). PCA analysis of the genotypic data was performed in GCTA v1.92.4 beta [95]. LD analysis was conducted in PopLDdecay-3.40 [96]. LD heatmaps were drawn by LDBlockShow v1.40 [97]. KEGG enrichment was performed using KOBAS 3.0 [98]. Visualization of the SNP markers on chromosomes was drawn by MG2C_v2.1 [99]. Correlation network of candidate marker genes and traits was constructed using Cytoscape v3.10.2 [100].

## Supplementary Material

Web_Material_uhaf144

## Data Availability

The genome resequencing data of this article can be found in the China National Center for Bioinformation National Genomics Data Center (https://ngdc.cncb.ac.cn/) under accession number CRA009145. Other data are provided in the article and supplementary data files.
