# Supplementary material for: GWAS identifies a molecular marker cluster associated with monoterpenoids in grapes
Source: Hortic Res. 2025 Jun 9;12(9):uhaf144. doi: 10.1093/hr/uhaf144 (PMC12313343; doi:10.1093/hr/uhaf144)
Supplement: Web_Material_uhaf144 [file web_material_uhaf144.zip › Supplementary Fig.S1-S12-R3.pdf]

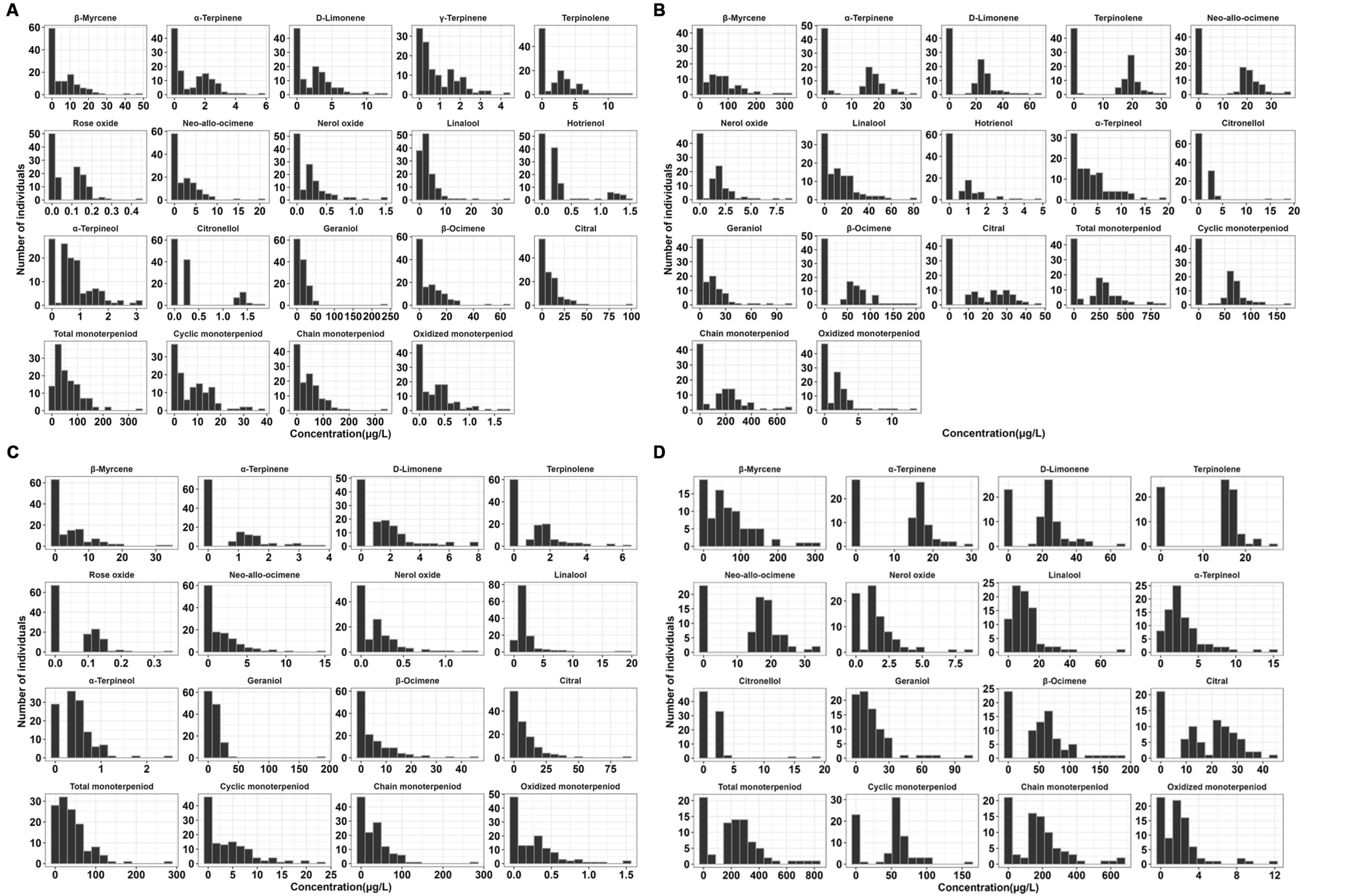

Figure S1. Concentration distribution patterns of monoterpenoid traits within the F1 population. The monoterpenoid traits presented in the figure include individual compounds, chain monoterpenoid, cyclic monoterpenoid, oxidized monoterpenoid and total monoterpenoid. Distributions of the total concentration of various traits in 2017 (A) and in 2018 (B). Distributions of the glycosidic concentration of various traits in 2017 (C) and in 2018(D).

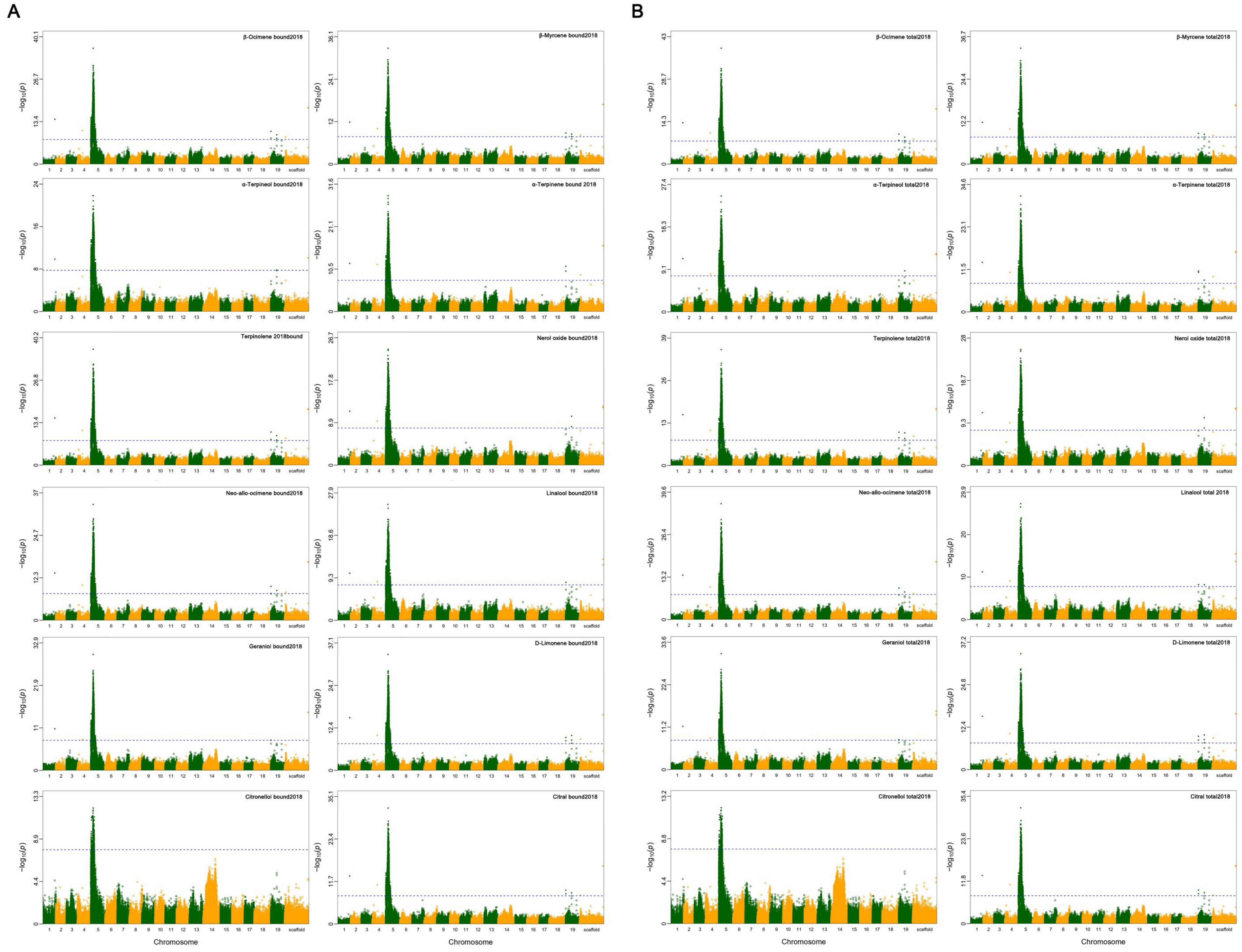

Figure S2. Manhattan plots of monoterpenoid traits in year 2018.

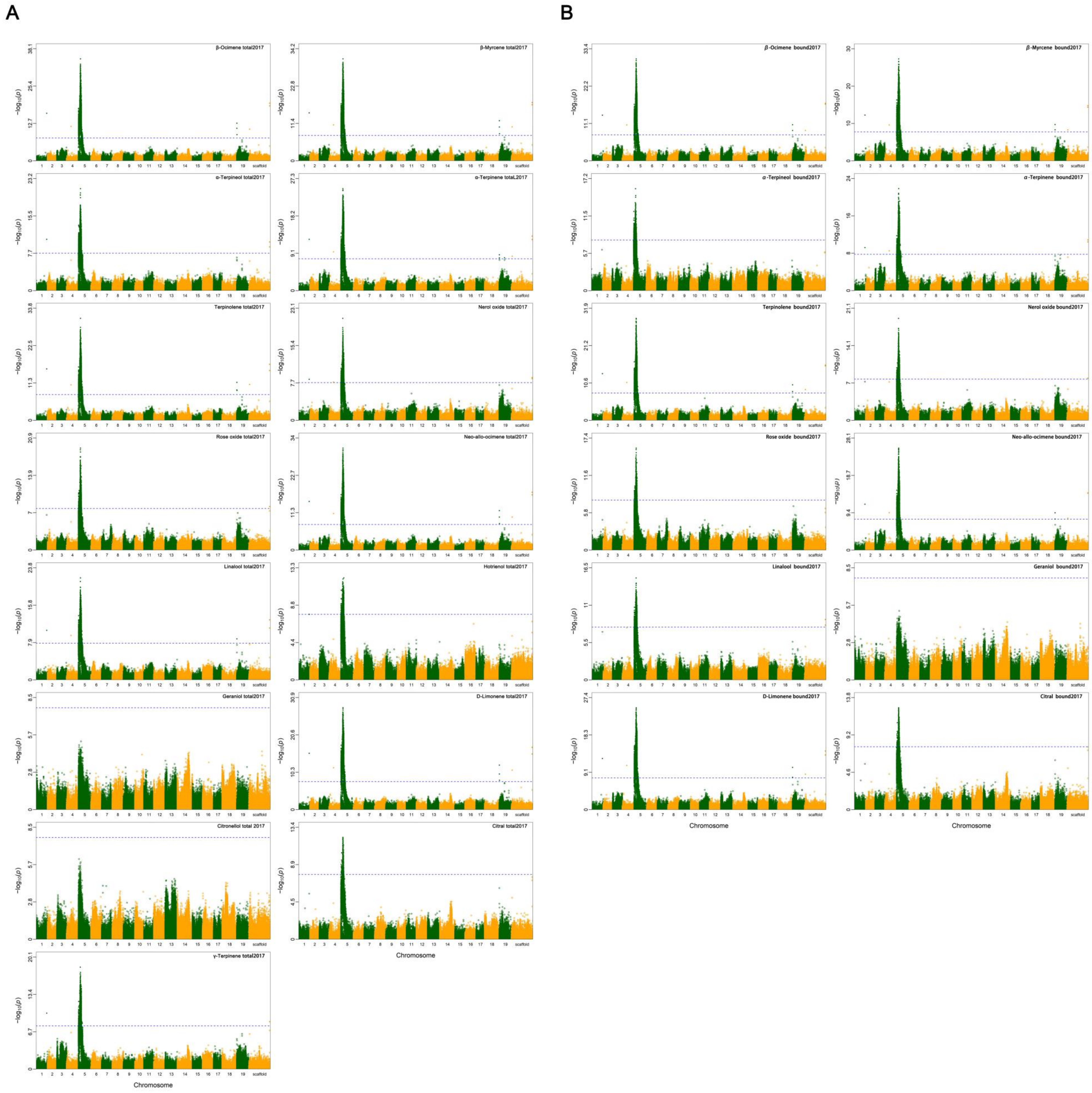

Figure S3. Manhattan plots of monoterpenoid traits in year 2017.

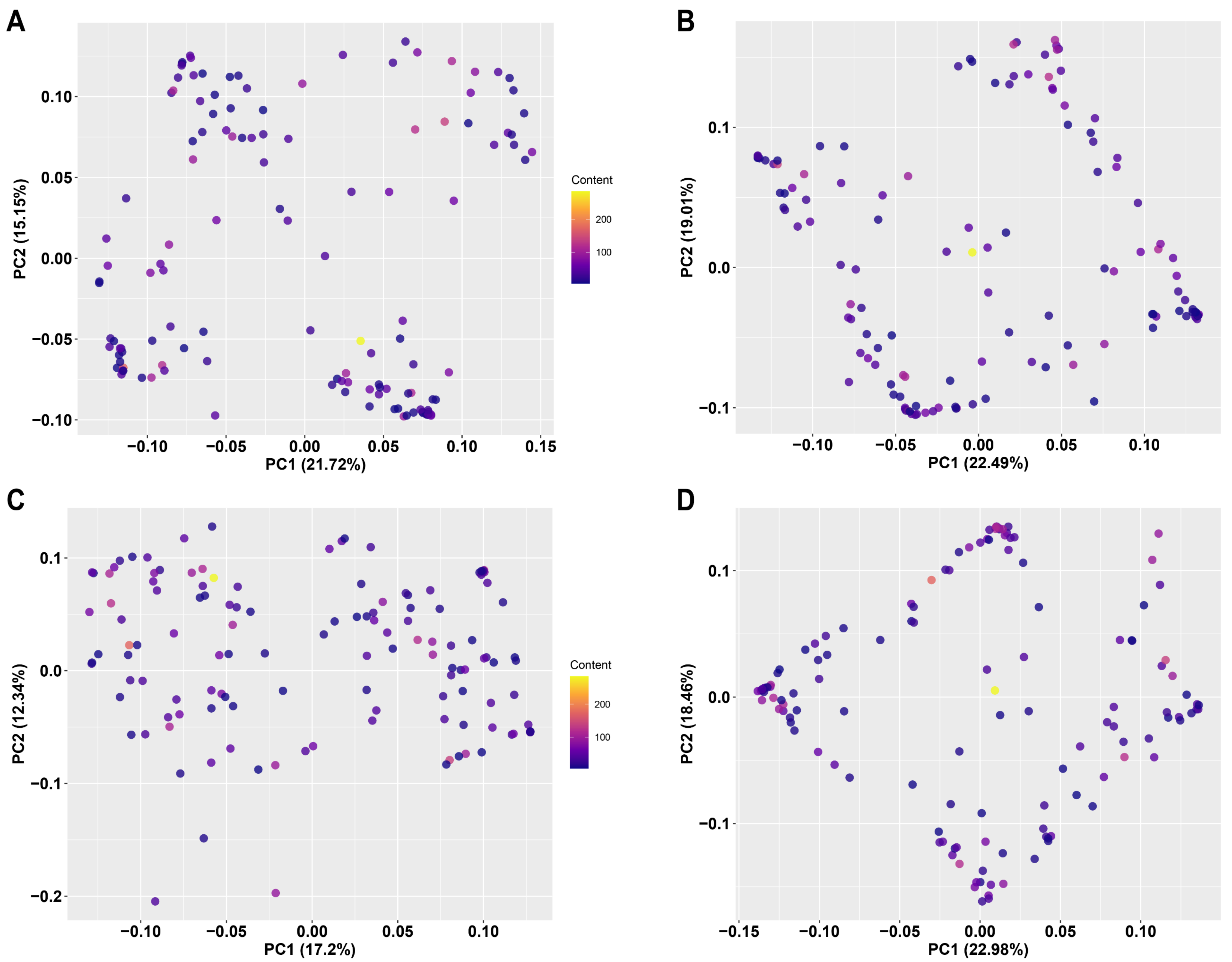

Figure S4. PCA plot of the genetic data on chromosome 4 (A), 9 (B),18 (C) and 19 (D). Points in the plot represent F1 individuals within the hybrid population. The glycoside content of total monoterpenoid (μg/L) in 2017 was used as a representative of phenotypes.

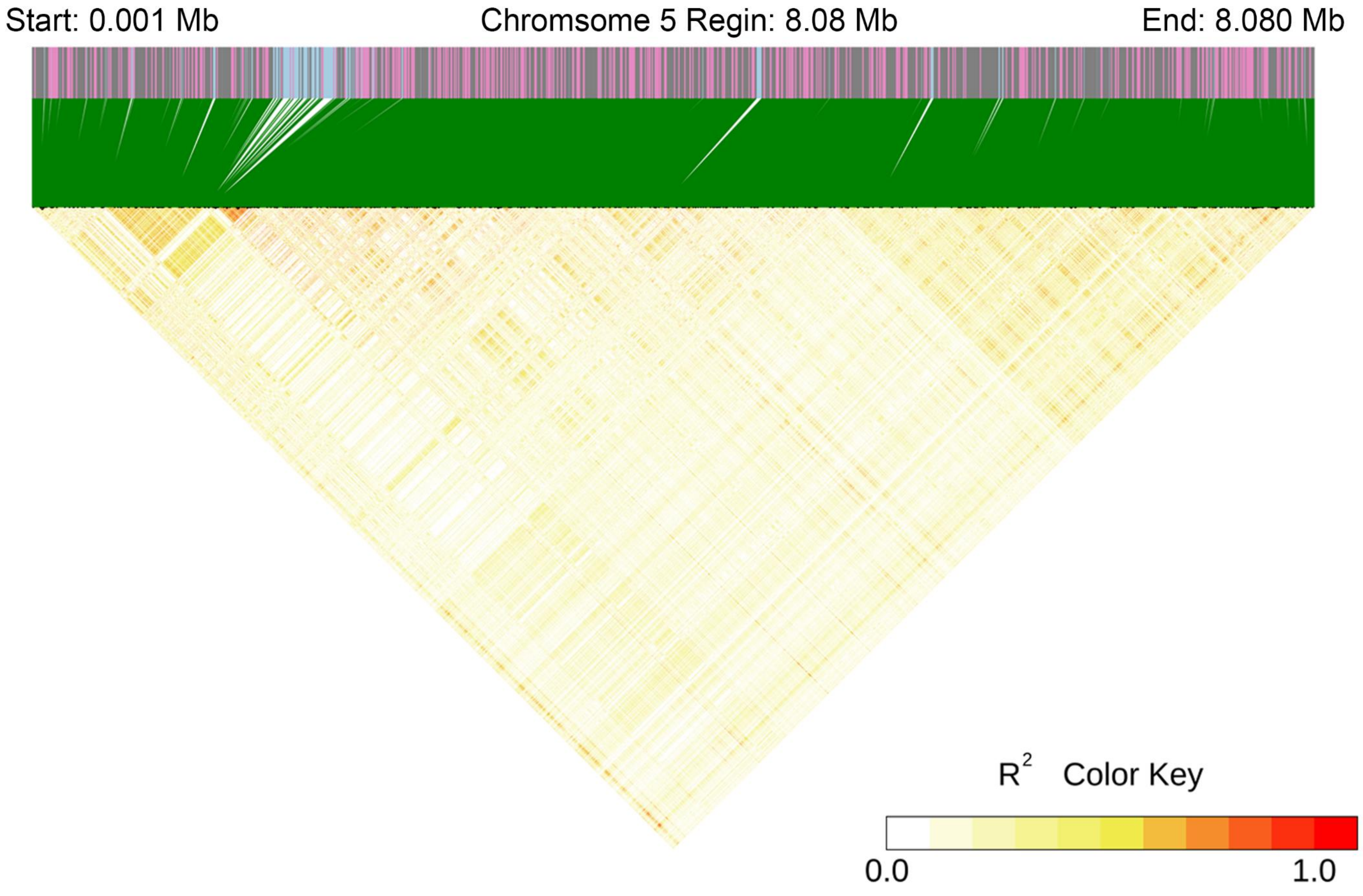

Figure S5. Linkage disequilibrium (LD) heatmap of the whole candidate interval on chromosome 5.

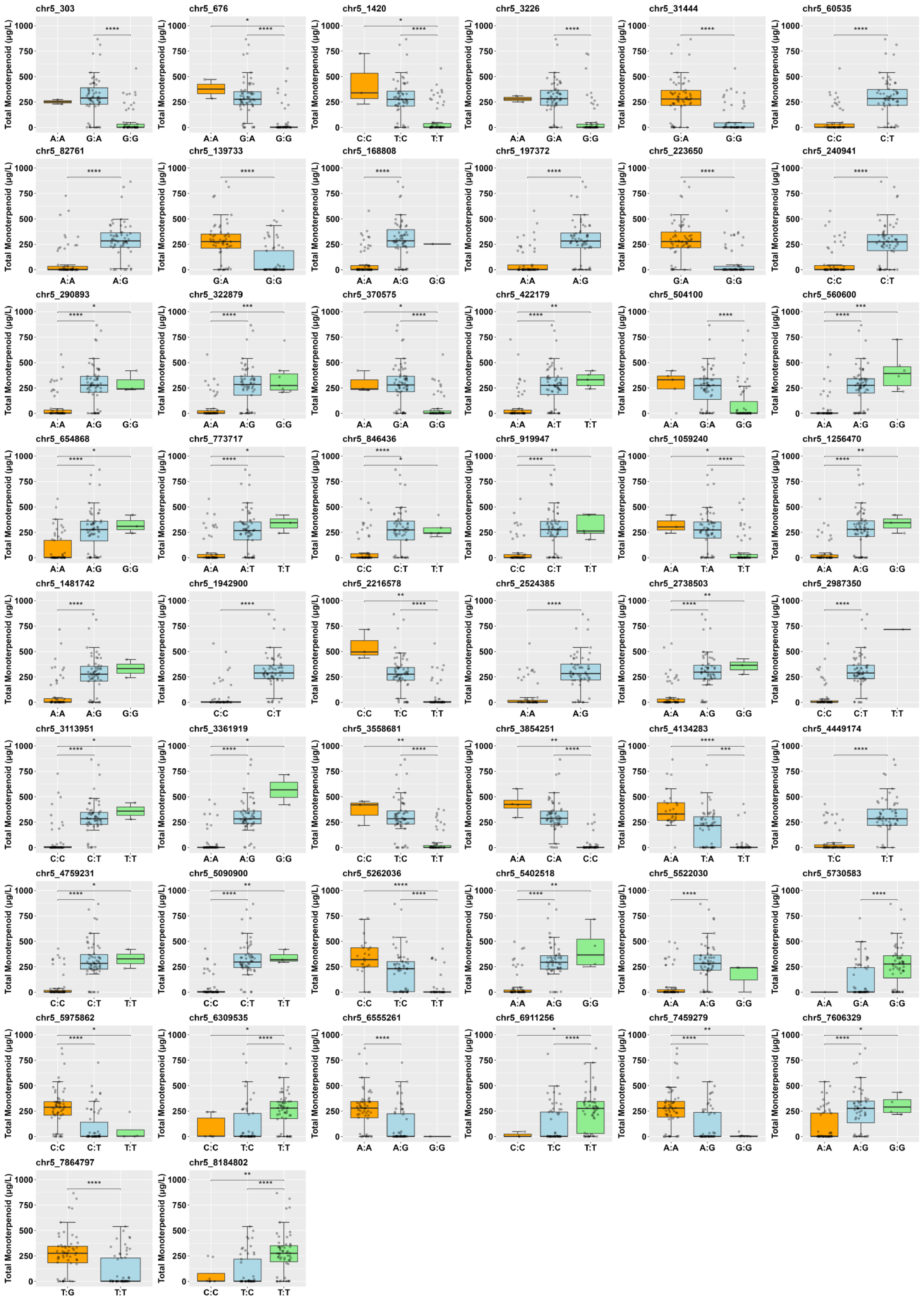

Figure S6. Concentration distribution box plots of different genotypes at 50 random selected sigSNPs on chromosome 5 in the F_1 population. The total concentration of monoterpenoids (μg/L) in 2018 was used as phenotypic representation. Asterisks denote kruskal test significance: *$p < 0.05$; **$p < 0.01$; ***$p < 0.001$; ****$p < 0.0001$.

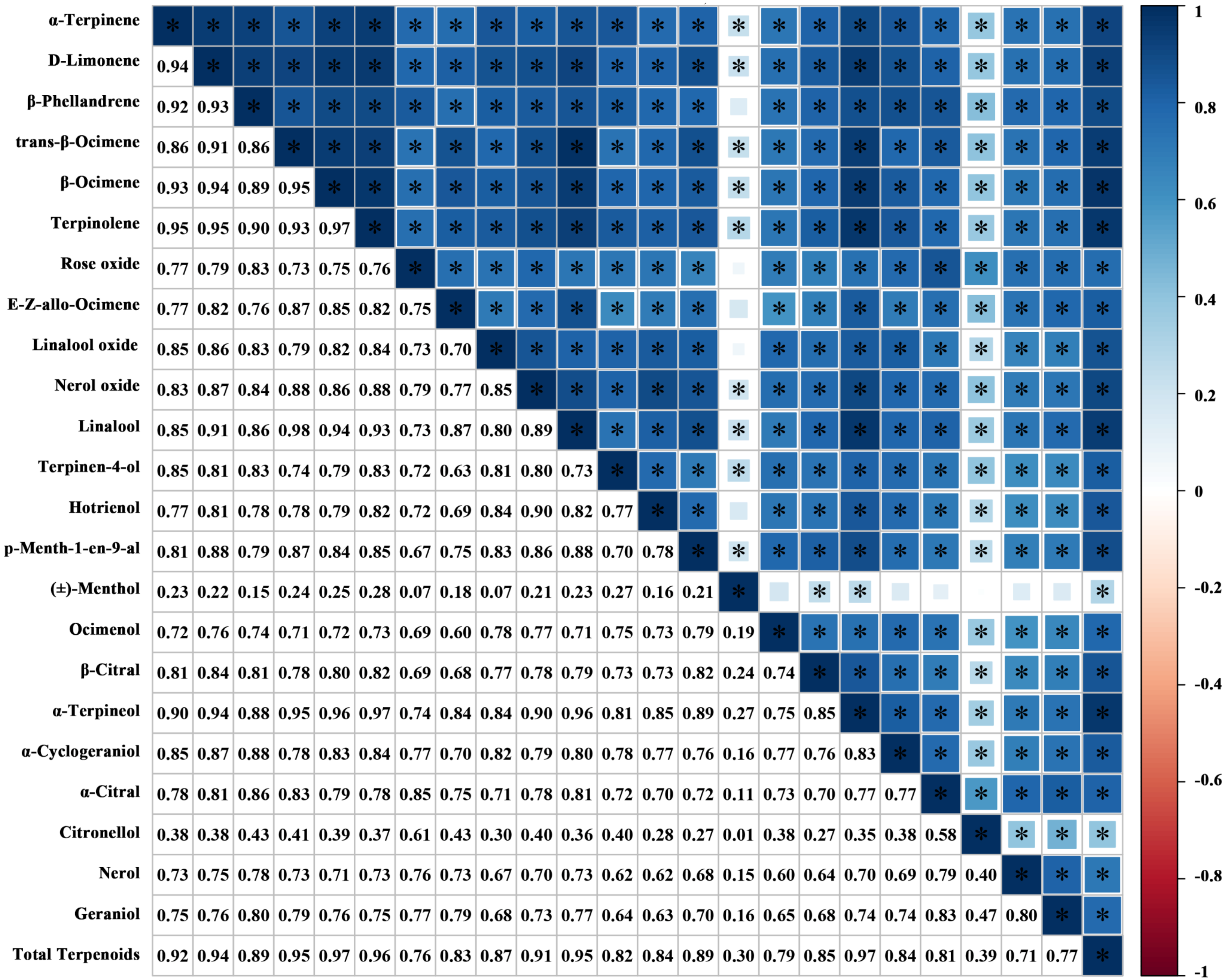

Figure S7. Correlation matrix of monoterpenoid traits in the germplasm population. The lower of each matrix shows the Spearman correlation coefficients, range from-1 (red) to 1 (blue); the * in the upper part shows the significant correlation ($p < 0.05$) and the color gradient is consistent with the value of coefficient.

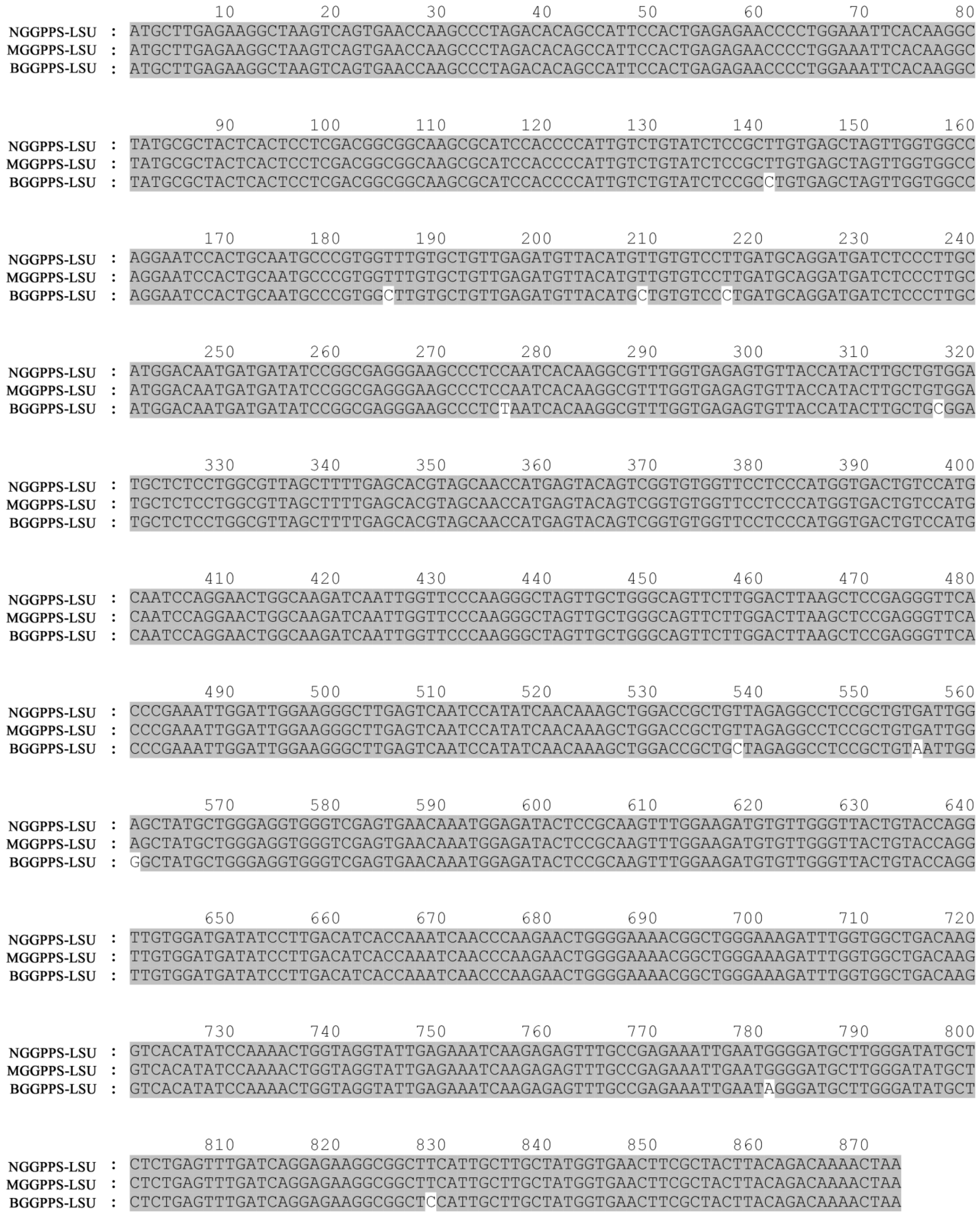

Figure S8. Coding sequence alignments of VvGGPPS-LSU from the PN40024reference genome (N), ‘Manseng Petit Blanc’ (M) and ‘Muscat Blanc 455’ (B).

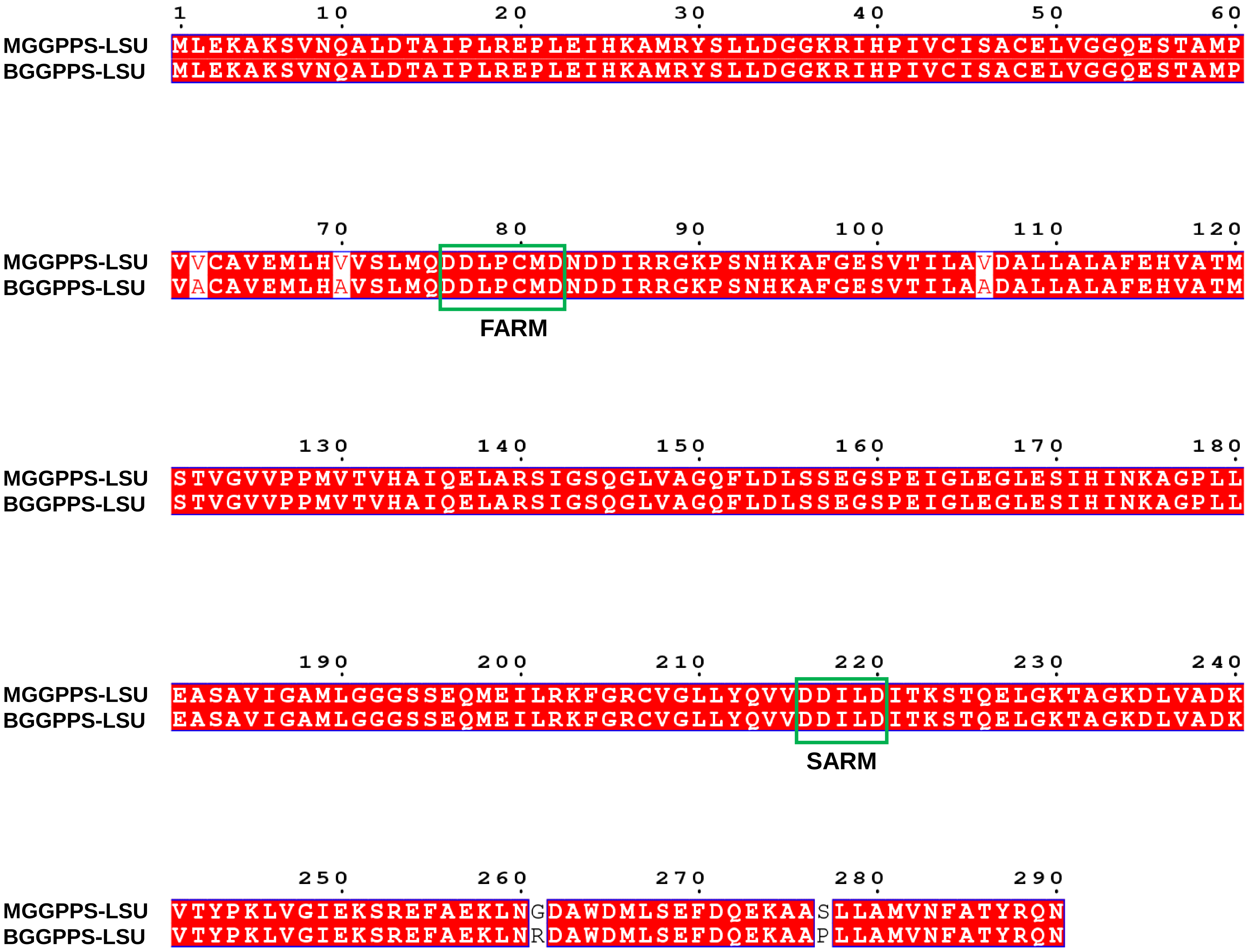

Figure S9. Amino acid sequence alignments of VvGGPPS-LSU of ‘Manseng Petit Blanc’ (M) and ‘Muscat Blanc 455’ (B).

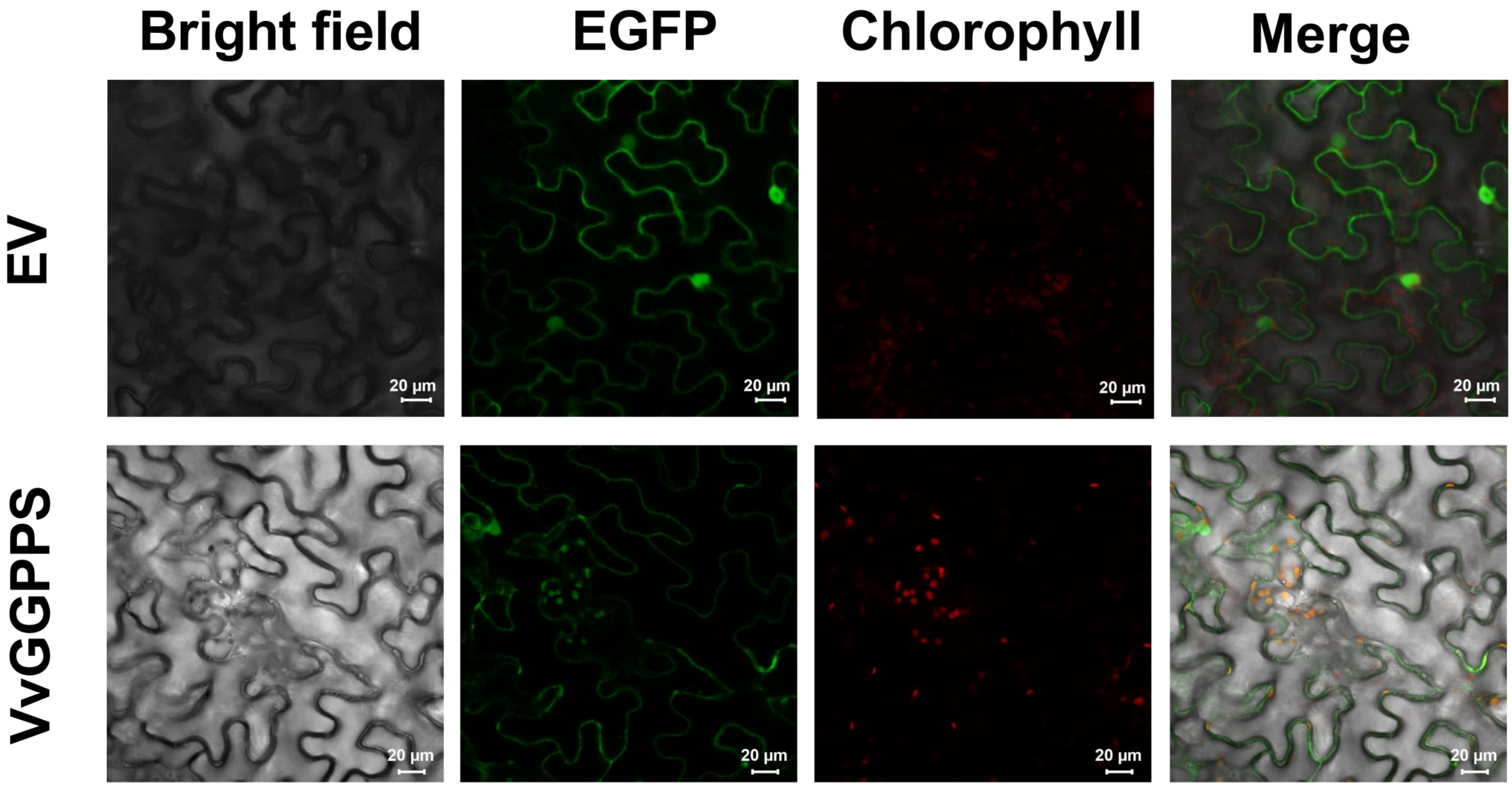

Figure S10. Subcellular localization of VvGGPPS-LSU.

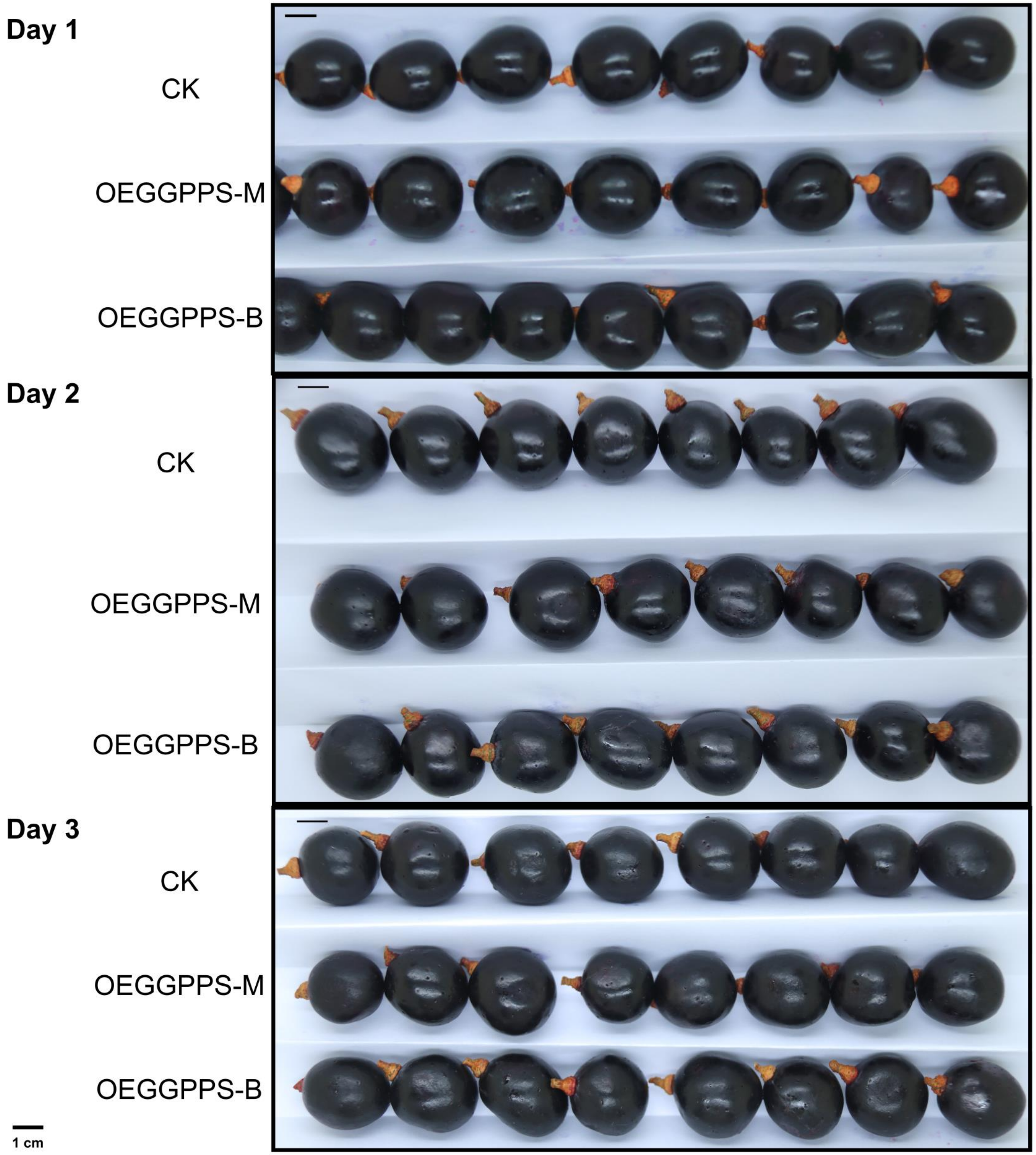

Figure S11. Image of the ‘Summer Black’ grapes in the three days after transient overexpression.

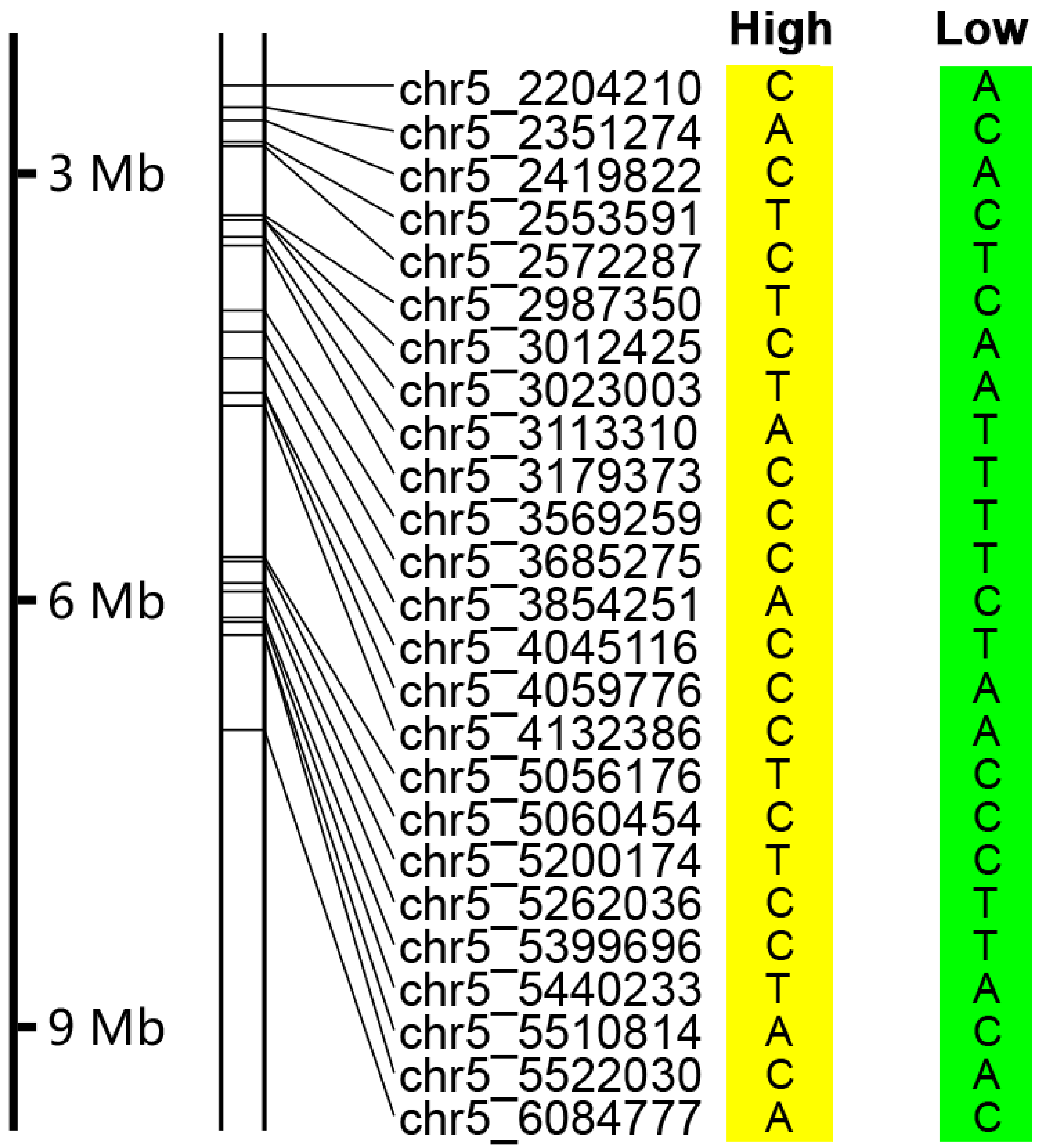

Figure S12. Marker sets composed of the 25 SNPs verified in the germplasm population, for indicating high and low monoterpenoid content in grapes.
